# Identification and validation of a ferroptosis-related gene to predict survival outcomes and the immune microenvironment in lung adenocarcinoma

**DOI:** 10.1186/s12935-022-02699-4

**Published:** 2022-09-24

**Authors:** Biao Deng, Jing Xiang, Zhu Liang, Lianxiang Luo

**Affiliations:** 1 Graduate School, Guangdong Medical University, Zhanjiang, 524023 Guangdong China; 2 Department of Cardiothoracic Surgery, Affiliated Hospital of Guangdong Medical University, Zhanjiang, 524023 Guangdong China; 3 The Marine Biomedical Research Institute, Guangdong Medical University, Zhanjiang, 524023 Guangdong China; 4 The Marine Biomedical Research Institute of Guangdong Zhanjiang, Zhanjiang, 524023 Guangdong China

**Keywords:** Ferroptosis, Lung Adenocarcinoma, RRM2, Tumor Microenvironment, Immunotherapy

## Abstract

**Background:**

Lung adenocarcinoma (LUAD) is a leading cause of cancer-related death worldwide. Ferroptosis, a form of cell death characterized by iron-dependent lipid peroxidation. However, the involvement of ferroptosis in the regulation of immune cell infiltration and its immunotherapeutic efficacy in LUAD remain unclear.

**Methods:**

The Cancer Genome Atlas (TCGA) LUAD cohort was used to assess the survival prognosis of FRGs and construct a seven-gene risk signature. Correlation tests, difference tests, and a cluster analysis were performed to explore the role of FRGs in the immune microenvironment and their immunotherapeutic efficacy in LUAD. The effects of FRGs on LUAD cells were assessed by Western blot, iron assay, and lipid peroxidation assay.

**Results:**

The seven-gene risk signatures of patients with LUAD were established and validated. FRG clustering based on 70 differentially expressed FRGs was associated with the immune microenvironment and indicated potential immune subtypes of LUAD. The seven-gene risk signature was an independent prognostic factor for LUAD and was used to divide the LUAD cohort into a high-risk and a low-risk group. Immunocyte infiltration levels, immune checkpoints, and immunotherapy response rates were significantly different between the two groups. Patients with high risk scores had lower overall levels of immunocyte infiltration but higher immunotherapy response rates. The key gene ribonucleotide reductase subunit M2 (RRM2) was associated with LUAD prognosis, which may be related to its ability to regulate the infiltration levels of activated mast cells and activated CD4 memory T cells. In addition, RRM2 was involved in ferroptosis, and its expression was up regulated in lung cancer tissues and the LUAD cell lines. Silencing RRM2 can inhibit the proliferation and induce ferroptosis of H1975 cells suggesting that silencing RRM2 could promote ferroptosis in H1975 cells.

**Conclusion:**

Our results revealed RRM2 as a promising biomarker and therapeutic target associated with tumor immune infiltration in patients with LUAD.

**Supplementary Information:**

The online version contains supplementary material available at 10.1186/s12935-022-02699-4.

## Introduction

Pulmonary carcinoma is the most serious malignancy, representing the leading cause of cancer deaths in developed and developing countries worldwide [1]. Lung adenocarcinoma (LUAD) is the most common histological type of non-small cell pulmonary carcinoma [2], and compared with other lung cancer subtypes, LUAD is more closely associated with genomic changes and is more heterogeneous [3]. Although mortality from pulmonary carcinoma has decreased in recent years, it still causes more deaths than breast, prostate, colorectal, and intracranial tumors combined [4]. More than half of patients with LUAD are diagnosed at advanced stages or with metastatic disease [5], and the patients have a poor prognosis with a low 5-year survival rate [6].

Ferroptosis, a recently discovered form of programmed cell death characterized by iron-dependent lipid peroxidation, is associated with a variety of diseases, particularly cancer; therefore, modulating the occurrence of ferroptosis in cancer cells could be a potential strategy for cancer therapy [7]. According to the literature, mitochondrial-induced cysteine starvation, endoplasmic reticulum-related oxidative stress, lysosome dysfunction, and lipid peroxidation related to Golgi stress contribute to the induction of ferroptosis in LUAD [8]. Researchers have also identified numerous genes involved in the organelle changes that induce ferroptosis. The regulatory mechanisms of these ferroptosis-related genes (FRGs) in the occurrence and development of pulmonary carcinoma have attracted increasing attention [9]. Bufu Tang et al. found that FRGs affected pulmonary carcinoma progression and tumor immunocyte infiltration. Ribonucleotide reductase subunit M2 (RRM2), a rate-limiting protein for deoxynucleoside triphosphate (dNTP) synthesis, inhibitedM1 macrophage polarization and promoted M2 macrophage polarization. However, ferrostatin-1 treatment effectively rebalanced macrophage polarization mediated by RRM2 inhibitors [10]. Huang et al. reported that aldo–keto reductase family 1 member C1 (AKR1C1) induced ferroptosis through multiple pathways and was associated with various cancer-infiltrating immune cells [11]. A study by Min Wang et al. showed that long noncoding RNA (lncRNA) and competing endogenous RNA (ceRNA) networks also play important roles in tumorigenesis and ferroptosis. Endogenous microRNA 6852 (miR-6852) inhibited cell growth, and by acting as an miR-6852 sponge, LINC00336 increased the expression of cystathionine-β-synthase (CBS), thereby inhibiting ferroptosis [12]. In addition to chromosomal genes, ferroptosis-related lncRNAs in the cytoplasm can also inhibit cancer by activating the p53 pathway. For example, studies by Chao Mao et al. showed that lncRNA P53RRA could bind to protein-binding protein 1(G3BP1) to activate G3BP1. The P53RRA–G3BP1 interaction replaced p53 in the G3BP1 complex, causing more p53 to be retained in the nucleus and leading to cell cycle arrest, apoptosis, and ferroptosis [13]. Luo et al. summarized the regulatory mechanism of ferroptosis and the effect of ferroptosis on tumor cell metabolism and antitumor immunity [14].

However, the significance of FRGs in the immune microenvironment and their potential role in immunotherapy for LUAD remains unclear, and thus FRGs require further investigation. In this study, we identified seven FRGs closely related to the immune microenvironment of LUAD, of which RRM2 affected the prognosis of LUAD by regulating the infiltration levels of activated mast cells and activated CD4 memory T cells. The expression of RRM2 also affected the response rate to LUAD immunotherapy. We also demonstrated the expression RRM2 is elevated in lung cancer tissues and knockdown of RRM2 induces ferroptosis in lung cancer cells H1975.We propose potential immune subtypes of LUAD based on the FRG cluster analysis that may lead to improved treatment for LUAD.

## Materials and methods

### Obtaining FRGand gene expression data for LUAD

A list of FGRs was downloaded from the public database FerrDb (http://www.zhounan.org/ferrdb/); after the noncoding genes were deleted, 382 FRGs remained. Transcriptome data for LUAD in fragments per kilobase of sequence per million mapped reads (FPKM) format were downloaded from The Cancer Genome Atlas (TCGA) (https://ancergenome.nih.gov/). The data included a total of 56,530 genes from 594 samples (535 tumor samples and 59 paracancerous samples); 492 of the samples had complete clinical information, including the survival time, survival status, age, gender, stage, and tumor/node/metastasis (TNM) state. Data from the GSE310219 cohort (54,675 genes from 307 early LUAD samples, 292 of which had complete clinical information) were downloaded from the Gene Expression Omnibus (GEO) database (https://www.ncbi.nlm.nih.gov/geo/).The clinical data of patients with LUAD included in this study are shown in Table 1.

**Table 1:** Clinical characteristics of lung adenocarcinoma patients included in this study

Characteristic	TCGA-LUAD (n = 492)	GSE310219 (n = 292)
Age (%)		
≤ 65	235(47.8)	179 (61.3)
> 65	257(52.2)	113 (38.7)
Gender (%)		
Male	228 (46.3)	249 (85.3)
Female	264 (53.7)	43 (14.7)
Stage (%)		
I + II	386 (78.5)	–
III + IV	106 (21.5)	–
T(%)		
T1 + T2	427 (86.8)	235 (80.5)
T3 + T4	62 (12.6)	52 (17.8)
Tx	3 (0.6)	5 (1.7)
N(%)		
N0	317 (64.4)	198 (67.8)
N1 + N2 + N3	165 (33.5)	93 (31.8)
Nx	10 (2.0)	1 (0.3)
M(%)		
M0	328 (66.7)	281 (96.2)
M1	25 (5.1)	8 (2.7)
Mx	139 (28.2)	3 (1.1)

### Differentially expressed genes (DEGs) between LUAD tissues and adjacent tissues

The mRNA data for the TCGA-LUAD and GSE310219 cohorts were standardized. The transcription sequences were then compared with the FRGs, which were analyzed using limma (an R software package). Differentially expressed genes (DEGs) with a log2 absolute value (FC) of > 1and an adjusted P-value of < 0.05 were selected.

### Establishment and validation of a risk signature

Univariate Cox regression analysis was used to screen genes related to overall survival (OS), and multivariate Cox regression analysis was used to identify genes with independent prognostic effects. A P-value of < 0.05was considered statistically significant. The genes screened using multivariate Cox regression were used to construct a risk signature; subsequently, the following predictive risk-scoring model was established combining the regression coefficient (β) with the gene expression level: RiskScore = (β1 * expression level of ALOX12B) + (β2 * expression level of DDIT4) + (β3 * expression level of SLC7A5) + (β4 * expression level of TRIB3) + (β5 * expression level of IL33) + (β6 * expression level of RRM2) + (β7 * expression level of CAV1). The establishment of the risk signature was achieved using the R software packages ‘survival’ and ‘survminer’.

### Prognostic potential analysis of risk signature

First, univariate and multivariate Cox regression were used to analyze whether the prognostic indicators were independent of other traditional clinical features, such as age, gender, and TNM state. The hazard ratio (HR) and 95% confidence interval (CI)of each variable were measured, and a P-value of < 0.05 was considered statistically significant. Then, the patients with LUAD in TCGA and the GEO database were divided into a high-risk group and a low-risk group with the median risk score as the boundary. The difference in the OS between the high-risk group and the low-risk group was analyzed using the Kaplan–Meier (K–M) curve. The prognostic potential and significance of the risk model were tested by a receiver operating characteristic (ROC) curve, and the area under the curve (AUC) was used to evaluate the specificity and sensitivity of the model. This analysis was conducted using the ‘survive’ package in R, and the ‘ggDCA’ package was used for the decision curve analysis (DCA) to analyze the net benefit of the risk signature. P < 0.05 represented statistical significance.

### Enrichment analysis

Gene ontology (GO) and Kyoto Encyclopedia of Genes and Genomes (KEGG) enrichment analyses were performed using the R packages ‘clusterProfiler’, ‘org.Hs.eg.db’, and ‘enrichplot’ to explore the differences in various molecular mechanisms between the high- and low-risk groups. GO analysis included biological processes (BPs), cell components (CCs), and molecular functions (MFs).

### Construction of a predictive nomogram

Using the R packages ‘survival’ and ‘regplot’, all independent predictors of LUAD were identified and then combined with the survival information of patients with LUAD; nomograms were drawn to predict 1-,3-, and 5-year survival rates.

### Analysis of tumor microenvironment

First, the StromalScore, ImmuneScore, and EstimateScore of each LUAD sample were calculated by the estimation algorithm, and the differences between the high- and low-risk groups were analyzed. Second, transcriptome data were quantitatively converted into the absolute abundance of specific cell types, including immune cells and stromal cells, using seven algorithms: CIBERSORT, TIMER, CIBERSORT-ABS, QUANTISEQ, MCPCOUNTER, XCELL, and EPIC. Third, immunocytes with independent predictive significance for the prognosis of LUAD were identified by univariate and multivariate Cox regression analysis, and the correlation between the expression of each risk gene and the level of immunocyte infiltration was analyzed using a correlation test. Fourth, the TCGA-LUAD cohort was clustered according to the level of immunocyte infiltration, and the LUAD samples were divided into a high-infiltration group and a low-infiltration group. The differences in clinical characteristics between the two groups were compared using a difference test. Finally, the scores of 29 immune-related functions or pathways were calculated by single set gene set enrichment analysis (ssGSEA), and the differences in scores between the high- and low-risk groups were compared (statistical significance was defined as P < 0.05 for all tests).

### Correlation analysis of immunotherapy

Tumor immune dysfunction and exclusion (TIDE) and microsatellite instability (MSI) scores of TCGA-LUAD participants were downloaded from the TIDE database (http://tide.dfci.harvard.edu/), and the tumor mutational burden (TMB) of patients with LUAD was downloaded using the R packages ‘TCGAbiolinks’ and ‘maftools’. Then, the Wilcoxon rank sum test was used to determine whether there were significant differences in the scores of five types of immunotherapies between the high- and low-risk groups. Moreover, the data of participants in the LUAD cohort (GSE126044) with anti-PDL1 treatment history were downloaded from the GEO database, and the differences in responses to anti-PDL1 immunotherapy between patients with high and low risk were compared. Additionally, the expression matrix of common immune checkpoints was extracted from the TCGA-LUAD cohort, and the expression differences between the high- and low-risk groups were compared. Finally, the correlation between first-line targeted therapy driving genes and risk genes was analyzed using a correlation test. P < 0.05was considered statistically significant.

### Drug screening

The risk genes were compared with the reference data set of the CMap database (https://portals.broadinstitute.org/cmap/), and the drugs were ranked according to the enrichment of risk genes in the reference gene expression profile. The drugs that significantly inhibited the expression of high-risk genes were screened for those with a P-value of < 0.05 and an enrichment score of < 0.The chemical structures of the selected drugs were searched in the PubChem database (https://pubchem.ncbi.nlm.nih.gov/).Using the ‘pRRophetic’ algorithm, a ridge regression model was constructed to predict the IC50sof these drugs according to the Genomics of Drug Sensitivity in Cancer (GDSC) (https://www.cancerrxgene.org/) cell line expression profile and TCGA gene expression profile, and antitumor drugs with significantly lower IC50s for high-risk LUAD samples were screened.

### Cell culture and transfection

The normal lung cell line BEAS-2B and seven lung cancer cell lines (H1299, A549, H460, H23, H838, PC-9, and H1975) were purchased from the American Type Culture Collection (ATCC) and maintained in 1640 medium containing 10% fetal bovine serum (FBS) (Gibco, Grand Island, NY, USA) and 1% penicillin–streptomycin (Gibco, Grand Island, NY, USA) and cultured at 37 °C with 5% CO_2_. The siRNA of RRM2 was purchased from Sangon Biotech (Shanghai, China) was used to silence the expression of RRM2. In this study, the sequence of RRM2 siRNA was sense: 5'- GCGAUUUAGCCAAGAAGUUTT-3'. H1975 cells were seeded in 12-well plates at a density of 6 × 10^4^ cells/well and transfected 24 h later. RRM2 siRNA or negative control (NC) siRNA was transfected to a final concentration of 20 nM using an siRNA transfection reagent (Polyplus, France). Finally, the transfected cells were detected after48h.

### Western blot

Proteins were extracted from cells, and cell lysates were prepared using RIPA lysate with phenylmethylsulfonyl fluoride (PMSF) (Solarbio, Beijing, China). Protein quantification was performed using a BCA protein assay kit (Sangon Biotech, Shanghai, China). Proteins were then separated by 12% sodium dodecyl sulfate–polyacrylamide gel electrophoresis (SDS–PAGE) and transferred to nitrocellulose membranes. The membranes were blocked with 5% bovine serum albumin (BSA) for 1 h and then incubated with primary antibody overnight at 4 °C. The next day, after washing the membrane three times with tris-buffered saline with Tween 20 (TBST), the membrane was incubated for 1 h at room temperature with horseradish peroxidase-labeled secondary antibody (1:4000), followed by three washes with TBST. Finally, the colors were developed using BeyoECL Moon (Beyotime Biotechnology, Shanghai, China).

### Detection of lipid peroxidation and apoptosis

C11-BODIPY 581/591 (10 μM; aBclone, Wuhan, China) was added to the H1975 cells, and the cells were incubated at 37 °C and 5% CO_2_ for 1 h. Then, the cells were washed twice with phosphate-buffered saline (PBS). After trypsin digestion, the cells were resuspended with PBS containing 5% FBS and analyzed by flow cytometry. An apoptosis detection kit (BD Biosciences) was used to prepare fluorescent dyes containing propidium iodide (PI) and fluorescein isothiocyanate (FITC) according to the manufacturer’s instructions. The H1975 cells were incubated in the dark at room temperature for 15 min and then analyzed by flow cytometry.

### Iron assay

FerroOrange (Dojindo, China) was used to detect the ferrous level of the cells. Following the manufacturer’s instructions, cells were incubated with FerroOrange for 0.5 h. The fluorescence intensity was evaluated using a rotating disk super-resolution laser confocal microscope.

### Immunohistochemical staining

From January 2020 to January 2021, 12 samples of LUAD tissues and normal tissues in the Department of Cardiothoracic Surgery, Affiliated Hospital of Guangdong Medical University, were selected. The obtained tissue was transferred to a −80 °C refrigerator in an ice box for subsequent immunohistochemical (IHC) experiments. All included research participants were assessed by experts in the pathology department of our hospital, and none of the patients studied had received radiotherapy, chemotherapy, targeted therapy, or immunotherapy before surgery. All patients signed informed consent before surgery. The tissue was fixed with 4% paraformaldehyde and embedded in paraffin to prepare slices with a thickness of 5 μM. The slices were then dewaxed with xylene and dehydrated in gradient concentrations of alcohol. Subsequently, 0.01 M sodium citrate (pH 6.0) was used for antigen repair, and endogenous peroxidase was blocked by adding 0.3% hydrogen peroxide (H_2_O_2_) and incubating in 10% goat serum albumin for 30 min. The slices were then incubated overnight with RRM2 primary antibody at 4 °C.The next day, the samples were incubated with 3,3′diaminobenzidine (DAB) after incubating with HRP-conjugated anti-rabbit secondary antibody for 1 h. The sections were stained with Mayer hematoxylin, dehydrated, removed with xylene, sealed with neutral resin, and detected by a multi-functional microporous microscope (BIOTEK).

### Statistical analysis

All data were generated, processed, and analyzed in R (version 4.1.1), R-Studio, and Strawberry Perl (5.32.1.1). Student’s t-test was used to determine the difference between the two groups, and the Wilcoxon rank sum test was used to identify differences for data from non-paired groups. P < 0.05 indicated that the difference was statistically significant. *P < 0.05, **P < 0.01, and ***P < 0.001.

## Results

### Obtaining DEGs of LUAD

**Fig. 1:**
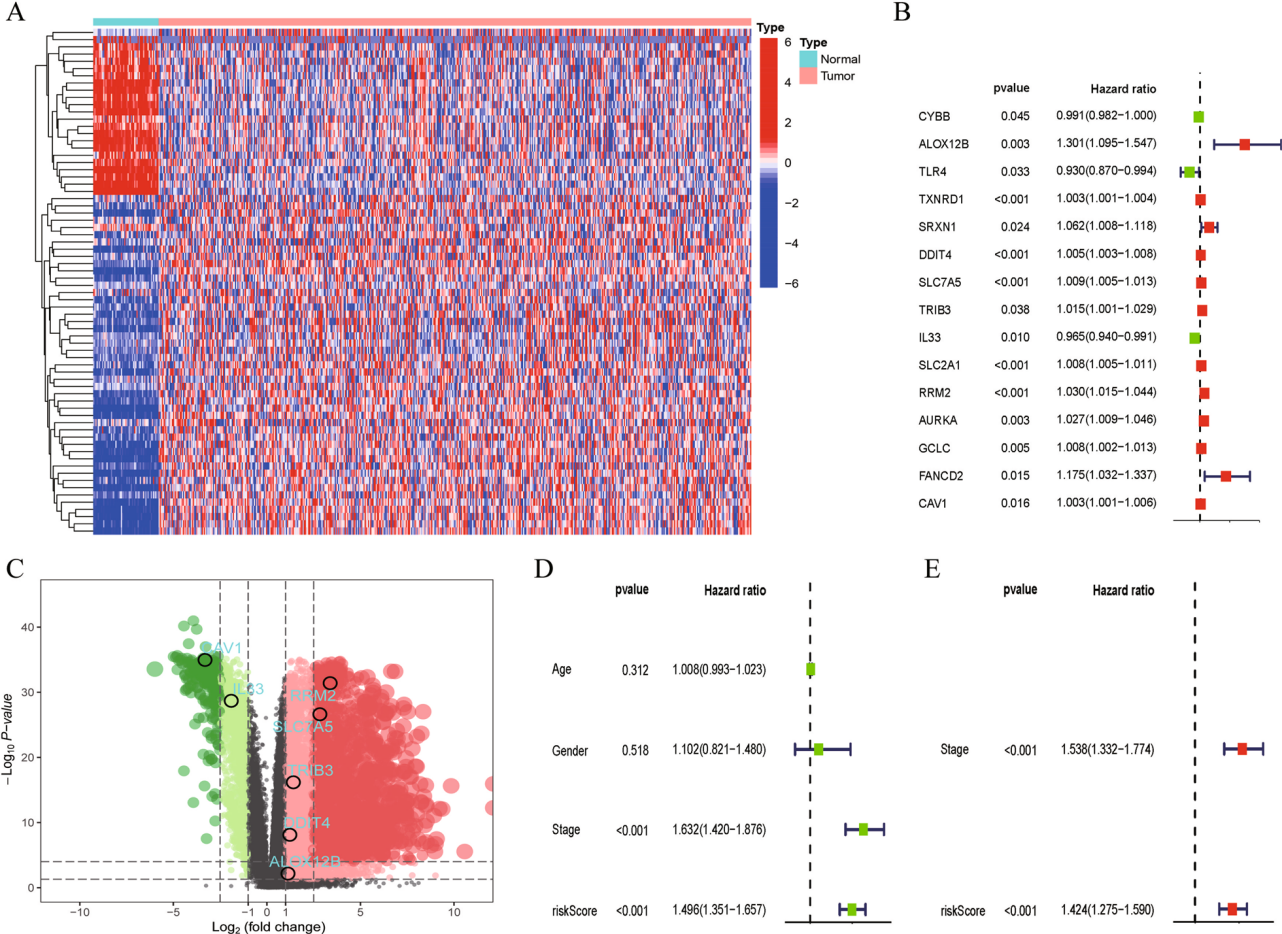
Construction of 7–gene Risk Signature. **A** The heatmap of FRDEGs between normal and tumor samples. **B** The forest plot of univariate Cox regression. **C** Differential expressed volcanic maps. The positions of seven key FRGs in the volcanic map. **D**, **E** Forest plots show independent prognostic roles of RiskScore and other clinical features

A total of 382 FRGs were downloaded from the public database FerrDb, and noncoding genes were deleted from the data set. Difference analysis showed that a total of 70 FRGs were differentially expressed between tumors and normal tissues; the characteristics of these FRGs in the TCGA-LUAD cohort are shown in Fig. 1A. In addition, all LUAD samples were clustered by consensus clustering based on 70 differentially expressed FRGs, and the best clustering effect was achieved when K = 4 (Additional file 1: Figure S1A–D). Surprisingly, significant differences were observed in OS between the four clusters (Additional file 1: Fig. S1E).

### Construction and validation of prognostic model

**Fig. 2:**
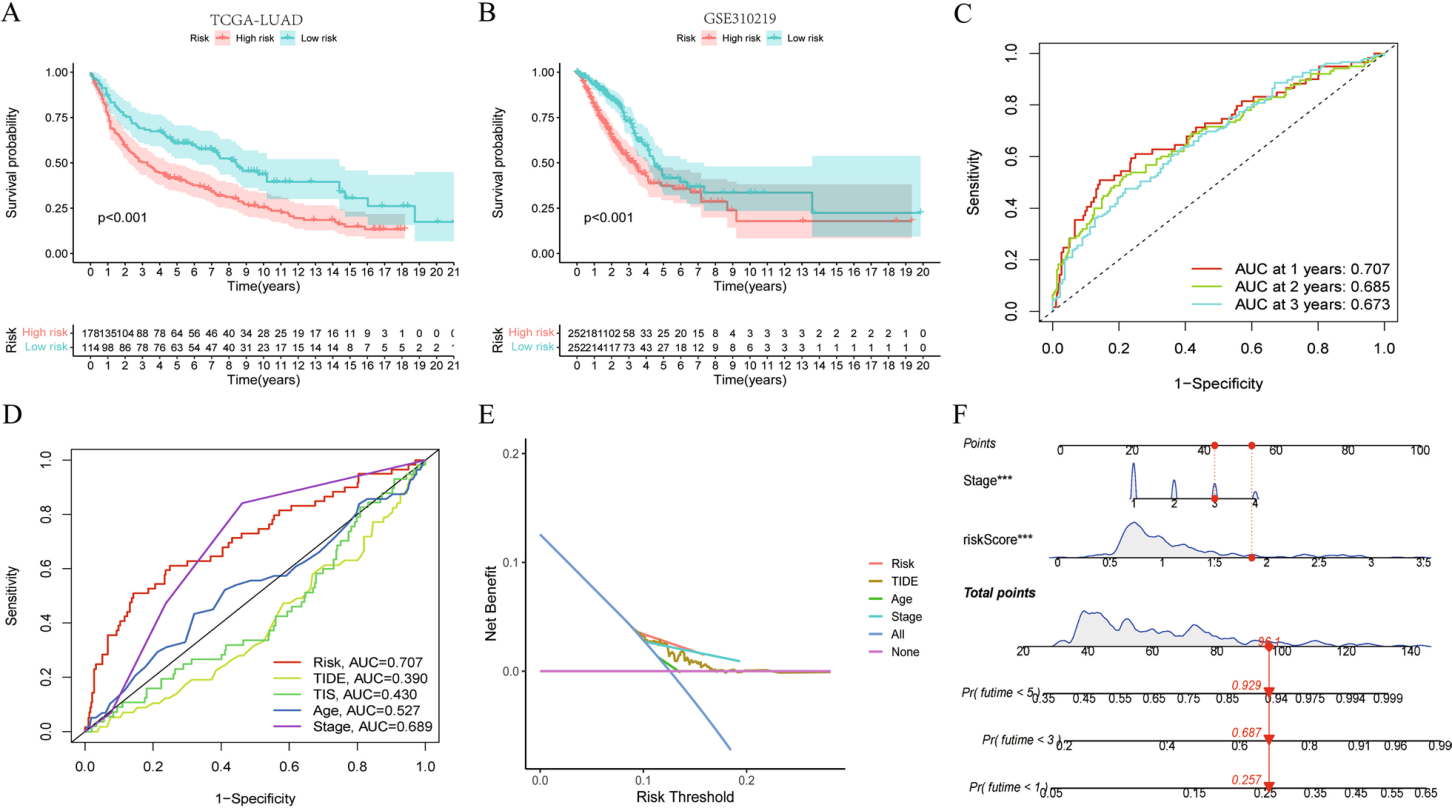
Verification of 7-gene Risk Signature and Construction of Nomo Graph. **A**, **B** Survival analysis between the high- and low-risk groups based on OS. **C** ROC curve of the 7-gene signature model. **D** 1-year survival rate predicted by 7-gene signature and other clinical features was compared in ROC curve. **E** Decision curve. **F** Nomogram for predicting 1,3,5-year survival rates in LUAD patients

As shown in the forest map, 15 DEGs associated with LUAD prognosis were identified by the R software package ‘survival’ in the TCGA cohort (Fig. 1B). Subsequently, seven FRGs with independent prognostic significance for patients with LUAD were obtained by multivariate Cox regression analysis on the 15 appealed DEGs; the locations of these FRGs in the differential volcanic map are shown in Fig. 1C. The Kaplan–Meier curve revealed the survival differences between the seven FRGs at different levels (Additional file 1: Fig. S1F–L). Ultimately, we established a seven-gene risk signature using the multivariate Cox regression coefficient, modeled as follows: Risk score of each patient = (0.262297470303534 * expression level of ALOX12B) + (0.0055627086031226 * expression level of DDIT4) + (0.00646442374221167 * expression level of SLC7A5) + (− 0.0131510931059613 * expression level of TRIB3) + (− 0.0300191156801198 * expression level of IL33) + (0.015384279700975 * expression level of RRM2) + (0.0046423481143834 * expression level of CAV1). According to the risk index ranking of 504 patients in TCGA-LUAD, patients were divided into a high-risk group and a low-risk group, with the median risk index as the cutoff between high and low risk. An independent prognostic analysis of the clinical characteristics of LUAD (age, gender, stage, and risk score) was conducted (Fig. 1D, E) after screening out the clinical information. The risk score and clinical stage were independent factors affecting the prognosis of LUAD. The clinical correlation chart in Additional file 3: Figure S3A shows the proportion of patients with high and low risk scores at each stage. Further analysis showed that the OS rate of TCGA-LUAD participants in the high-risk group was significantly lower than the OS rate of those in the low-risk group (P < 0.001) (Fig. 2A). Figure 2B shows that a difference in the OS rate was also present between the low-risk and high-risk groups in the GSE30219 cohort; the OS rate of the high-risk group was significantly lower than that of the low-risk group (P < 0.001). The ROC curve (Fig. 2C) shows that our risk signature had high sensitivity and specificity in predicting the OS rate of patients with LUAD. The AUCs were 0.707, 0.685, and 0.673 for 1-, 2-, and 3-year OS, respectively. We then added clinical features, including TIDE, tumor inflammation signature (TIS), age, and stage, to compare the accuracy of OS prediction. As shown in Fig. 2D, the risk index was the best predictor of OS. The DCA (Fig. 2E) showed that the net benefit of the risk index was the largest. Finally, a nomogram based on the two independent prognostic factors was constructed to quantify the survival probability of patients with LUAD in 1, 3, and 5 years (Fig. 2F).

### Enrichment analysis

We conducted an enrichment analysis to explore the potential biological characteristics of 70 DEGs. The 25 pathways shown in Additional file 2: Figure S2A were enriched in the KEGG enrichment analysis. The more prominent pathways, represented by red bubbles, included fluid shear stress and atherosclerosis, HIF-1 signaling pathways, lipid and atherosclerosis, and ferroptosis. GO enrichment analysis showed that the biological processes (including responses to oxidative stress and metal ions) and the cell components (including the apical plasma membrane, cell apex, and oxidoreductase complex) were significantly enriched (Additional file 2: Figure S2B). Furthermore, GSEA enrichment analysis showed that the high- and low-risk groups were enriched incompletely different functions or pathways. Among high-risk patients, small cell pulmonary carcinoma, melanoma, cancer pathways, thep53 signaling pathway, and pathogenic *Escherichia coli* infection were significantly enriched (Additional file 2: Figure S2C).

### Difference of immune related indexes between LUAD patients

**Fig. 3:**
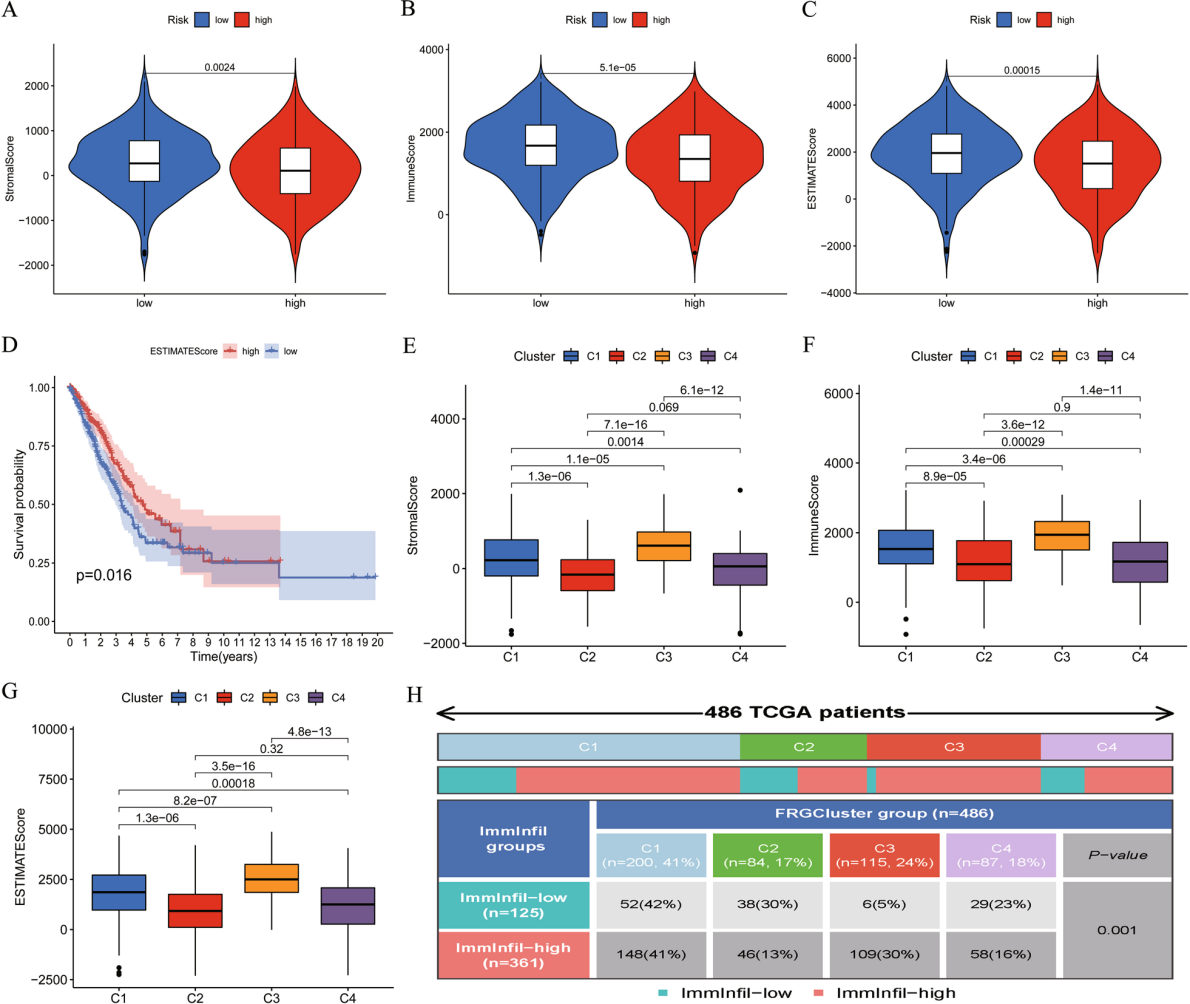
Difference of immune related indexes between LUAD patients. **A**, **B**, **C** Violin Chart. Differences in StromalScore, ImmuneScore and EstimateScore between high- and low-risk patients. **D** Different OS based on different levels of ESTIMATEScore. **E**, **F**, **G** Differences in StromalScore, ImmuneScore and EstimateScore among the four FRGs clusters. **H** Proportion of high- and low-infiltration of immunocytes in four FRGs clusters

We calculate the StromalScore, ImmuneScore, and EstimateScore for each LUAD sample usingthe estimate algorithm. The t-tests revealed significant differences in StromalScore, ImmuneScore, and EstimateScore between high- and low-risk groups (Fig. 3A–C). To explore the influence of different levels of immune-related scores on the prognosis of patients, the StromalScore, ImmuneScore, and EstimateScore of all patients with LUAD were divided into high- and low-score groups by average. The OS rates of the two groups were compared using the Kaplan–Meier method; Fig. 3D shows that a higher EstimateScore represented a better prognosis. The same was true for ImmuneScore and StromalScore (Additional file 5: Figure S5A, B). Significant differences in risk scores were also observed among the four clusters (Additional file 3: Figure S3C).

### LUAD immune subtype based on FRG clustering

**Fig. 4:**
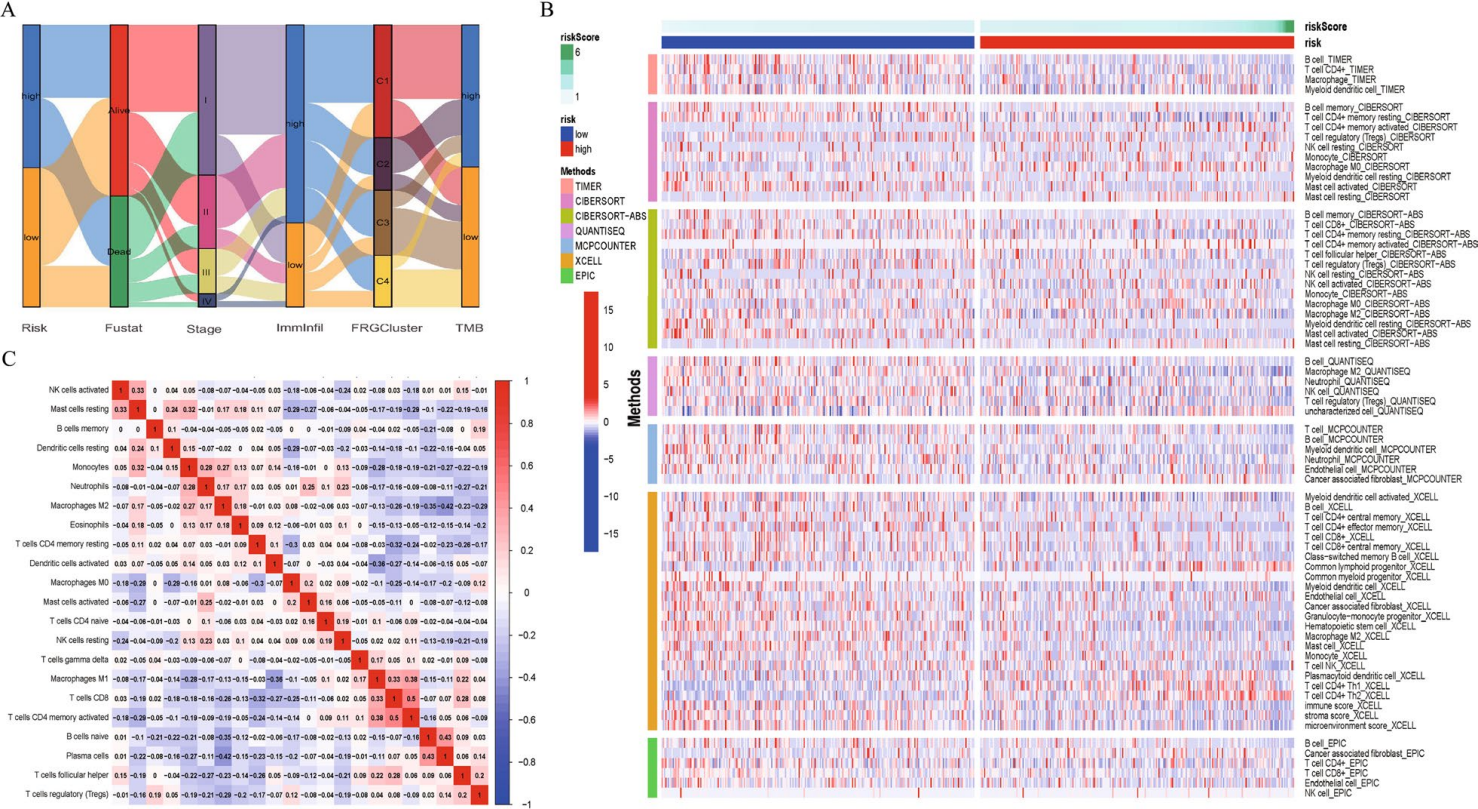
Difference and correlation of immunocyte infiltration in LUAD patients. **A** Alluvial diagram of the relationship between RiskScore and several features. **B** Immune cell infiltration heatmap, 7 algorithms to assess immune cell infiltration level among the high-and low-risk groups. **C** Correlation heatmap, correlation of infiltration levels of immune cells in LUAD

In addition, the t-test revealed significant differences in immune-related scores among the four clusters obtained by the consensus clustering analysis (Fig. 3E–G), suggesting that the four clusters obtained for70 differentially expressed FRGs may serve as immune subtypes. To explore the relationship between the aforementioned four clusters and the two groups clustered according to the level of immunocyte infiltration, we drew a clinical correlation heat map, shown in Fig. 3H. Significant differences were observed in immunocyte infiltration among the four clusters. For example, the samples with high immunocyte infiltration in the composition of C3 were significantly more than those with low immunocyte infiltration, whereas the samples with low immunocyte infiltration in the C2 and C4 clusters were more. The mulberry diagram in Fig. 4A shows the corresponding relationship between immune infiltration level, FRG cluster, risk level, and other clinical features. The above results show that the four clusters identified by the clustering of 70 FRGs may represent the immune subtypes of LUAD.

### Further analysis of immune cell infiltration in LUAD tumor microenvironment

The CIBERSORT algorithm was used to quantify the levels of 22 immune cells in each LUAD sample, and at-test was used to compare the levels of immune cells between the high-risk and low-risk patients. As shown in Fig. 4B, significant differences were observed in the levels of B cell memory, CD4 memory T cells, activated CD4 memory T cells, regulatory T cells (Tregs), resting NK cells, monocytes, M0 macrophages, resting dendritic cells, resting mast cells, and activated mast cells between high-risk and low-risk patients. The relative proportion of 22 immune cells in all LUAD samples is shown by a barplot (Additional file 3: Figure S3D). When Kaplan–Meier curves were used to compare the effects of immunocytes with different infiltration levels on the OS rates, we found that different infiltration levels of 12 typesof cells, such as activated CD4 memory T cells and resting mast cells, were associated with different OS rates (Additional file 4: Figure S4A–L). To learn more about the differences between immune cells in high- and low-risk patients, we analyzed the data using six other algorithms (TIMER, CIBERSORT-ABS, QUANTISEQ, MCPCOUNTER, XCELL, and EPIC); Fig. 4B shows the different infiltration levels of immunocytes in each database.

### Relationship between immunocyte infiltration and clinical characteristics

**Fig. 5:**
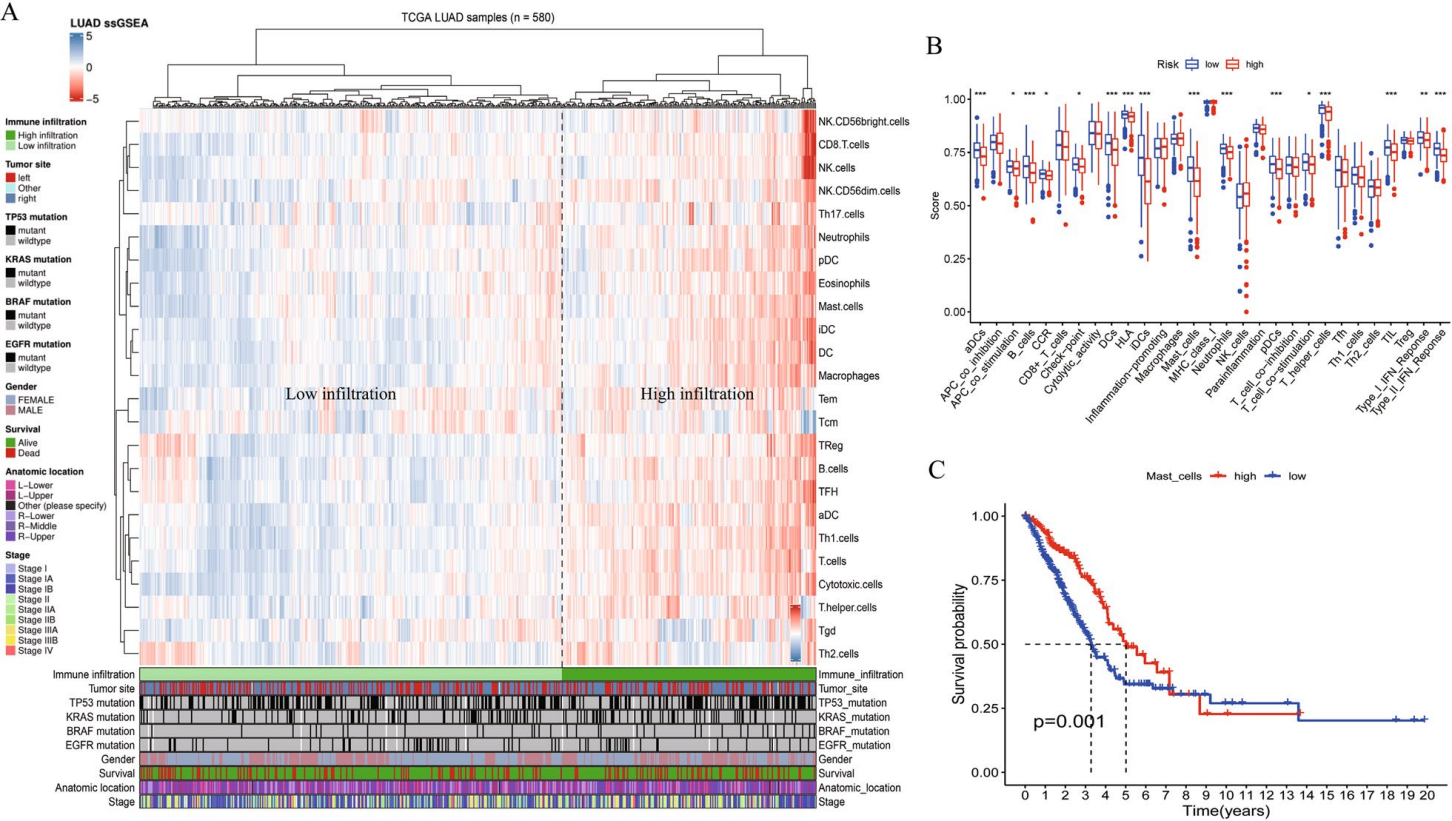
Analysis of Immunocytes Clustering and immune-related functions in LUAD Patients. **A** Clustering of immunocytes infiltration. TCGA-LUAD cohort was divided into two groups with high-and low-infiltration levels according to 24 immunocytes. **B** The difference of immune-related function scores between high- and low-risk groups. **C** Different infiltration levels of Mast-cell represent different LUAD OS

**Fig. 6:**
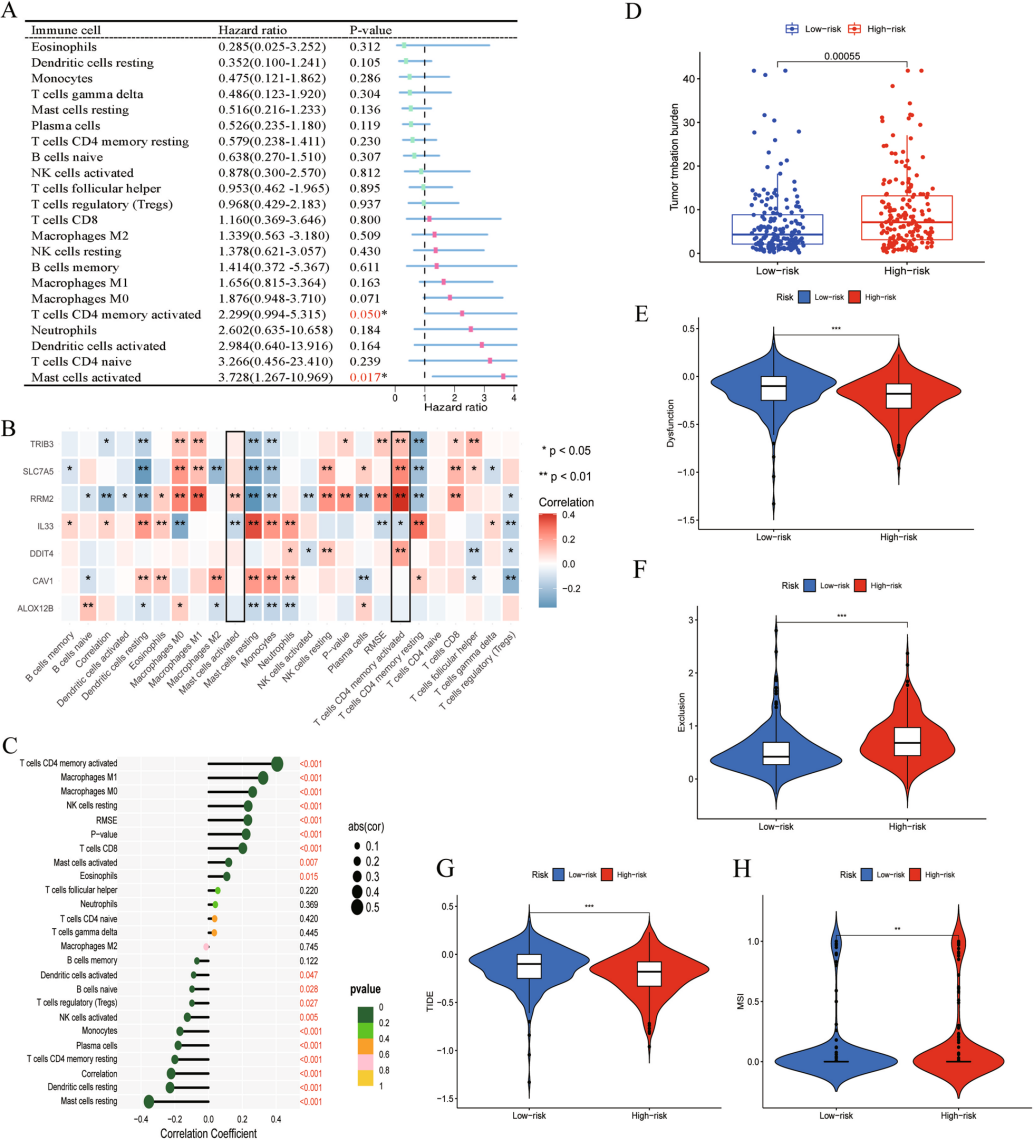
Analysis of the relationship between FRGs and prognosis-related immunocytes and immunotherapy-related indicators. **A** Immunocytes which play independent prognostic role in LUAD patients. **B** Correlation between the expression levels of 7 hug FRGs and the infiltration levels of immunocytes. **C** Immunocytes whose infiltration level correlated with RRM2 expression. **D** Difference in TMB among high- and low-risk groups; **E**, **F**, **G**, **H** Violin Chart. Differences in Four Immunotherapy Related scores between high- and low-risk Groups

The correlation heat map shown in Fig. 4C reveals the relationship between the immune cells; most modules are blue, suggesting that the immune cell infiltration in the LUAD microenvironment is negatively correlated. To understand the correlation between immune cell infiltration and clinical characteristics, we calculated the immune cell infiltration level of all LUAD samples in TCGA using the ssGSEA algorithm and then downloaded the clinical information for all LUAD samples from TCGA using the R package ‘TCGAbiolinks’. The 580 LUAD samples with clinical information were clustered into two groups according to the level of immune cell infiltration and visualized by a heat map (Fig. 5A). The left portion, dominated by blue, represents the samples with less immunocyte infiltration, whereas the right portion, dominated by red, represents the samples with higher immunocyte infiltration. The clinical information, such as survival status, stage, gender, tumor location, and related driving gene mutation state, corresponded to the heat map below. In addition, to better understand the immune differences between high-risk and low-risk groups, we quantified 29 immune-related functions using the ssGSEA algorithm. As shown in Fig. 5B, 16 immune-related functions or pathways, such as Adcs and CCR, showed significant differences. When the Kaplan–Meier curve was used to compare the effects of different levels of immune-related functions on OS rates, we found that various levels of multiple immune-related functions or pathways predicted different OS rates. Figure 5C shows that varying levels of mast cell-related immune functions or pathways corresponded to a different prognosis for patients with LUAD. After univariate Cox regression analysis, we identified activated mast cells and activated CD4 memory T cells as prognostic immune cells of LUAD; these two cell types, which had high levels of infiltration, were adverse prognostic factors for LUAD (Fig. 6A). Further analysis confirmed that the expression levels of seven risk genes significantly affected the infiltration level of immunocytes in the LUAD microenvironment, and the infiltration level of activated mast cells, which was most related to the prognosis of LUAD, was affected by the expression levels of RRM2 and IL33 (Fig. 6B). The expression of RRM2 not only regulates the infiltration of activated mast cells but is also related to the infiltration of 19 immunocytes, such as CD4 memory T cells, dendritic cells, and macrophages (Fig. 6C).

### Risk signature and immunotherapy

To explore the role of the risk signature in guiding immunotherapy, we identified16 patients with pulmonary carcinoma receiving anti-PDL1 immunotherapy in the GSE126044 cohort. Additional file 5: Figure S5G shows the differences in immunocyte infiltration and response to anti-PD-L1 immunotherapy between high-risk and low-risk patients. High-risk patients had lower immunocyte infiltration levels, but their response rates to anti-PD-L1 immunotherapy were higher. In addition, we found that the expression of RRM2 was higher in patients who had no response to anti-PDL1 treatment (P = 0.052) (Additional file 5: Figure S5F). We then downloaded the TIDE and MSI scores of the LUAD cohort from the TIDE database, and we downloaded the TMB data of patients with LUAD using the R packages ‘TCGAbiolinks’ and ‘maftools’. The Wilcoxon rank sum test revealed significant differences between the five immune therapy-related scores described above in high-risk and low-risk patients (Fig. 6D–H). The patients with LUAD with high TMB had worse OS (Additional file 5: Figure S5D). Subsequent correlation analysis showed that the risk score was positively correlated with TMB (R = 0.23, P = 0.000018) (Additional file 5: Figure S5C). The correlation heat map in Additional file 3: Figure S3B shows the distribution of clinical features in the high-risk and low-risk groups. The distribution of TMB, gender, stage, T, and N was significantly different in the high-risk and low-risk groups.

**Fig. 7:**
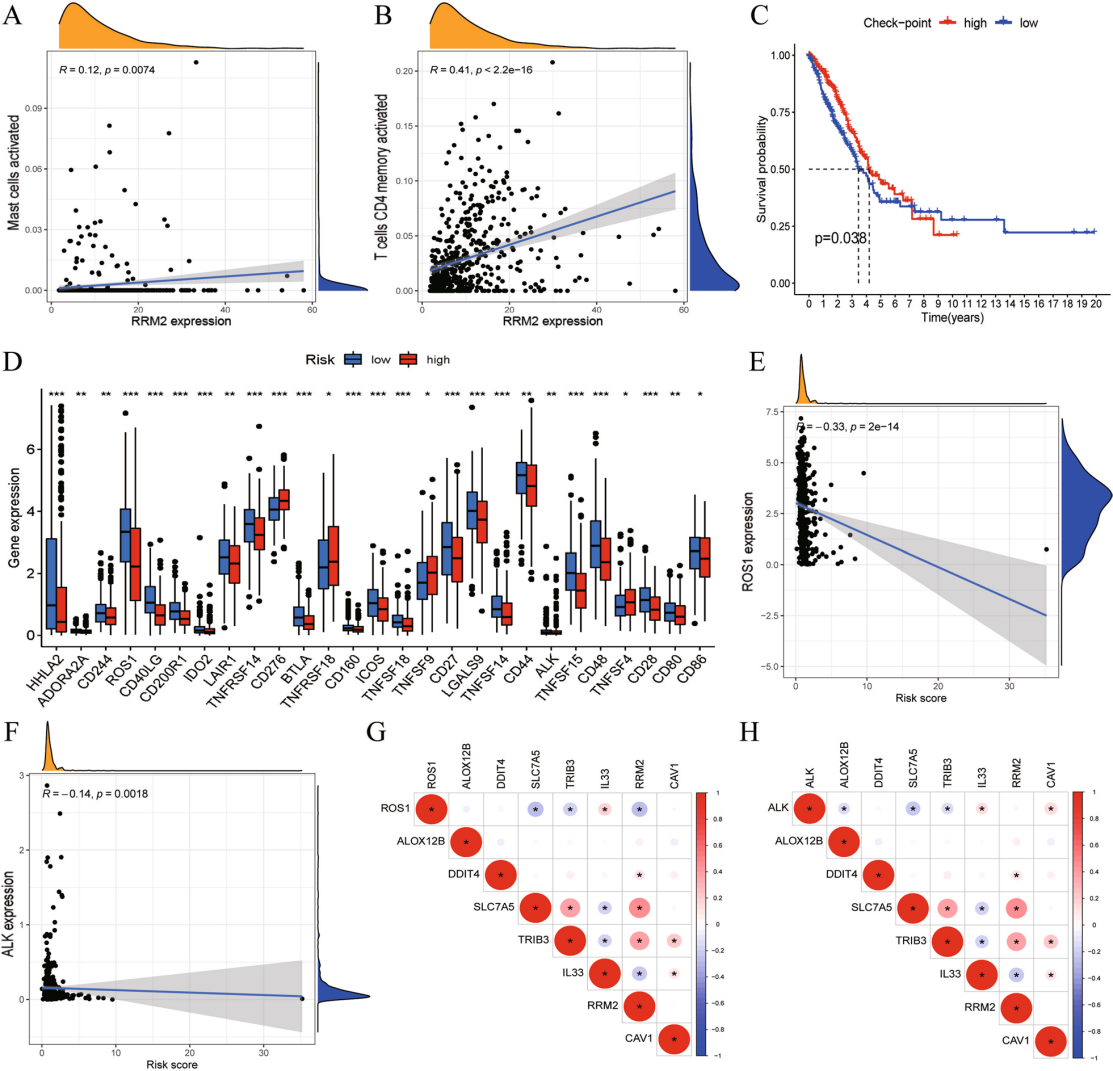
FRGs regulate the level of immunocytes infiltration and immune checkpoint expression in LUAD patients. **A**, **B** The change trend of the infiltration level of Mast-cells and T cells CD4 memory activated with the expression of RRM2. **C** Different Check-point levels represent different LUAD OS. **D** 27 common checkpoint genes are differentially expressed between high and low risk groups. **E**, **F** The expression of ROS1 and ALK change with risk score. **G**, **H** Correlation of the expression of ROS1 and ALK with 7 key FRGs

To study the relationship between the seven-gene risk signature and immunotherapy, we counted the mutations of seven key genes in patients with LUAD. As shown in the waterfall diagram in Additional file 5: Figure S5E, ALOX12B had the highest the mutation probability (18%), followed by RRM2 and IL33, and missense mutation was the most common form. To further explore the relationship between the expression of the seven risk genes and immunocyte infiltration, we conducted a correlation analysis, as shown in Fig. 7A, B. The expression level of RRM2 positively regulated the infiltration abundance of activated mast cells (R = 0.12) and activated CD4 memory T cells (R = 0.41), and the infiltration level of resting mast cells was significantly negatively correlated with the expression level of ALOX12B (R =  −0.13) (Additional file 3: Figure S3F). When the ssGSEA algorithm was used to analyze the scores of immune-related functions or pathways, the scores of the checkpoints between high- and low-risk LUAD samples were significantly different. The checkpoints were divided into high and low groups using the average, and the Kaplan–Meier curve showed significant differences in survival (Fig. 7C). Next, we analyzed the expression levels of 49 common immune checkpoints in high-risk versus low-risk patients, as shown in the box plot in Fig. 7D. The expression levels of ALK, ROS1, CD44, and other 27 checkpoints were different. As shown in Fig. 7E, F, the expression levels of ALK and ROS1 decreased as the risk score increased. Furthermore, the correlation analysis showed that ALK and ROS1 were significantly associated with key risk genes. The expression of ROS1 was positively correlated with that ofIL33 and negatively correlated with that ofSLC7A5, TRIB3, and RRM2 (Fig. 7G). The expression of ALK was positively correlated with the expression of IL33 and CAV1and negatively correlated with the expression of ALOX12B, SLC7A5, and TRIB3 (Fig. 7H). The expression of two additional checkpoints, PD-1 and PD-L1, were also significantly correlated with multiple prognostic FRGs (Additional file 5: Figure S5H, I).

### Overexpression of RRM2 in tumor tissues

**Fig. 8:**
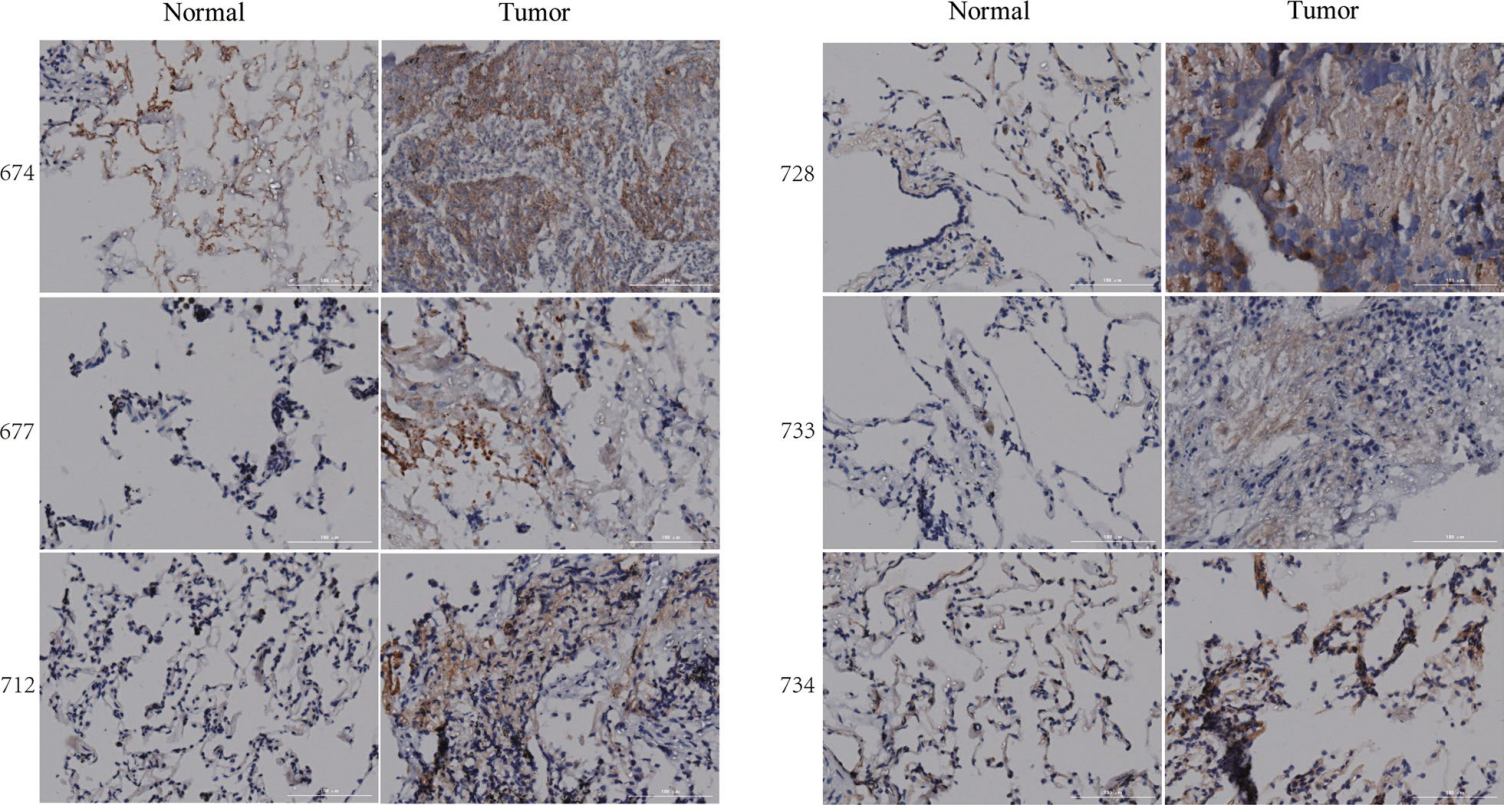
The expression of RRM2 in normal lung tissue and lung cancer tissue was detected by immunohistochemistry. 674, 677, 712, 728, 733 and 734 are patient numbers. Expression of RRM2 in LUAD tissues was higher than that in normal tissues

To detect the expression of RRM2 in normal lung tissues and lung cancer tissues, we performed immunohistochemical experiments for comparison. The results showed that the expression of RRM2 in tumor tissues was higher than that in normal lung tissues (Fig. 8).

### Silencing RRM2 induced ferroptosis in lung cancer cells

**Fig. 9:**
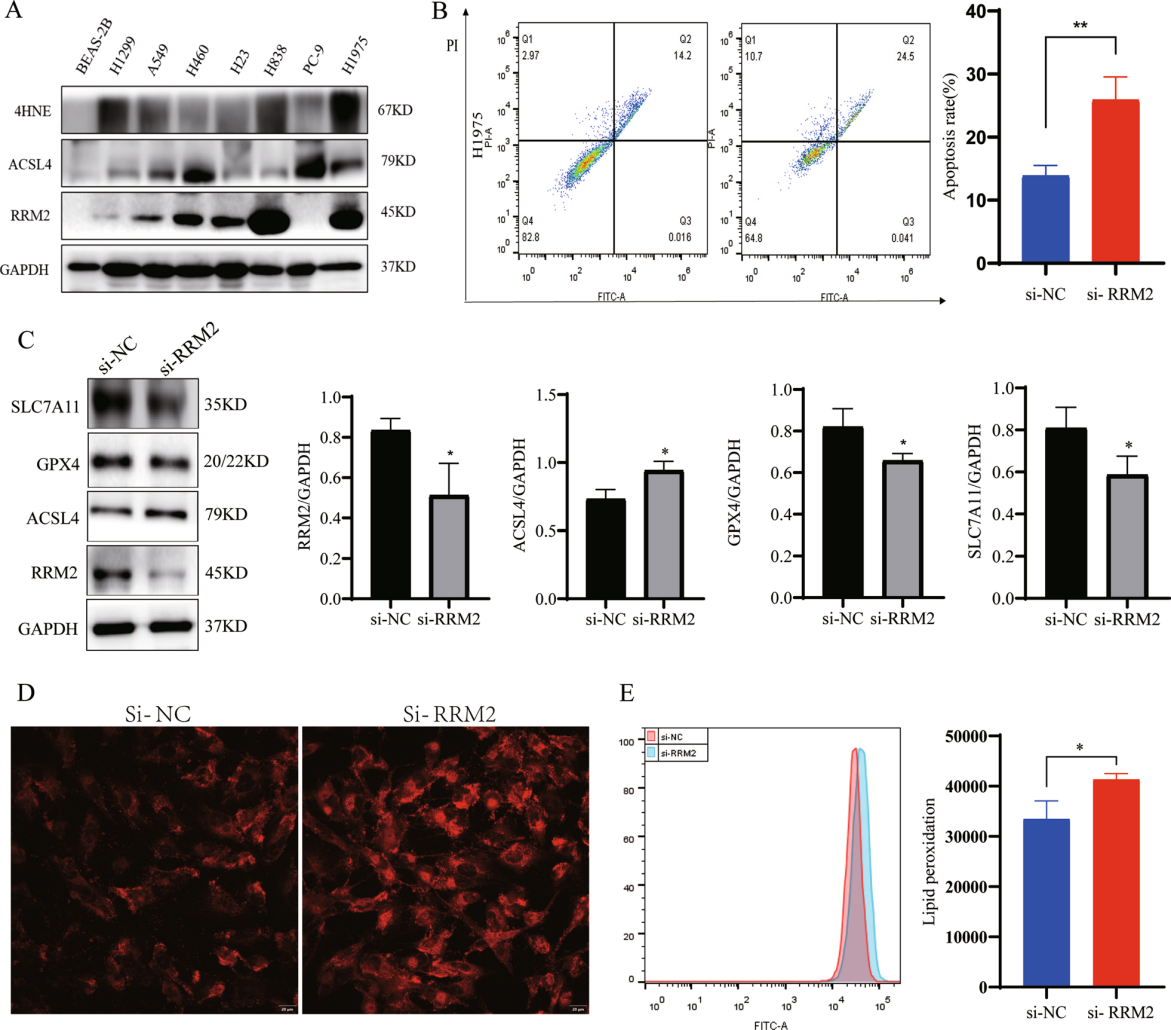
Effects of silencing RRM2 on ferroptosis. **A** Expression level of RRM2,4HNE and ACSL4 in different lung cancer cell lines by Western blotting. **B** The results of cell death level after silencing RRM2 by flow cytometry. **C** The expression of ferroptosis-related indicators was changed in H1975 cells after silencing RRM2. **D** The fluorescence intensity of cells after silencing RRM2 was evaluated using a rotating disk super-resolution laser confocal microscope. **E** Detection of cell lipid peroxidation level after silencing RRM2 by flow cytometry

Ferroptosis is a recently discovered form of cell death characterized by lipid peroxidation accumulation and iron dependence. Further study on the specific mechanism of ferroptosis is expected to bring new prospects for cancer treatment. To determine the clinical relevance of RRM2 expression, we detected RRM2 expression in a normal lung cell line (BEAS-2B) and seven lung cancer cell lines (H1299, A549, H460, H23, H838, PC-9, and H1975). Western blotting showed that RRM2 protein was highly expressed in H1975 cells. In addition, we detected the expression of 4HNE and ACSL4 in different lung cancer cell lines. The results showed that 4HNE and ACSL4 were highly expressed in H1975 cells (Fig. 9A), indicating that H1975 cells may be more sensitive to ferroptosis. Therefore, we used H1975 cells for subsequent experiments. After H1975 cells were transfected with RRM2siRNA for 48 h, cell death was detected by flow cytometry. The results showed that silencing RRM2 could induce cell death **(**Fig. 9B**)**. To further study the effect of silencing RRM2 on ferroptosis, Western blotting was used again to detect the expression of several proteins in H1975 cells with RRM2 siRNA transfected. The results showed that the expression of RRM2 in the silencing group was significantly decreased, indicating that silencing RRM2 was successfully. In the meantime, the expression of ferroptosis-related indicators like SLC7A11 and GPX4 were decreased, while ACSL4 were increased in H1975 cells after silencing RRM2 **(**Fig. 9C). Then, we continued to detect the levels of ferrous ion and lipid peroxidation in lung cancer cells after silencing RRM2. The results showed that silencing RRM2 could induce an increase in ferrous ion level **(**Fig. 9D**)** and lipid peroxidation accumulation **(**Fig. 9E**)** in H1975 cells. Ultimately, we demonstrated that silencing RRM2 induced ferroptosis in H1975 cells.

### Screening drugs for high-risk FRGs

To predict effective therapeutic drugs for patients with high-risk LUAD, we constructed a ridge regression model to predict the drug IC50using the GDSC cell line expression profile and TCGA gene expression profile; we screened six antitumor drugs with significantly lower IC50 in high-risk LUAD: bosutinib, dasatinib, gefitinib, tipifarnib, docetaxel, and JNK inhibitor VIII (Additional file 6: Figure S6A–F). These six antitumor drugs could kill tumor cells in patients with high-risk LUAD. In addition, we compared the risk genes with the reference data set of the CMap database and obtained the correlation of 1309 drugs according to the enrichment of risk genes in the reference gene expression profile. With P < 0.05 as the standard, 158 drugs were found to be significantly enriched in high-risk genes; of these, 86 drugs promoted the expression of high-risk genes, and 72 drugs inhibited the expression of high-risk genes. Medrysone, phenoxybenzamine, vorinostat, thioguanosine, apigenin, and chrysin were selected as the six drug candidates enriched with more prominent inhibitors of high-risk gene expression, and their chemical structures were found in the PubChem database (Additional file 6: Figure S6G-L). The detailed information on these six drugs is shown in Table 2.

**Table 2:** Six small molecule drugs for high-risk LUAD patients slected from CMAP database

Rank	CAMP name	Enrichment	P-value
5	Phenoxybenzamine	−0.924	0.00004
8	Thioguanosine	−0.914	0.0001
9	Apigenin	−0.906	0.00014
19	Medrysone	−0.73	0.00073
22	Chrysin	−0.913	0.00116
23	Vorinostat	−0.51	0.00192

## Discussion

**Fig. 10:**
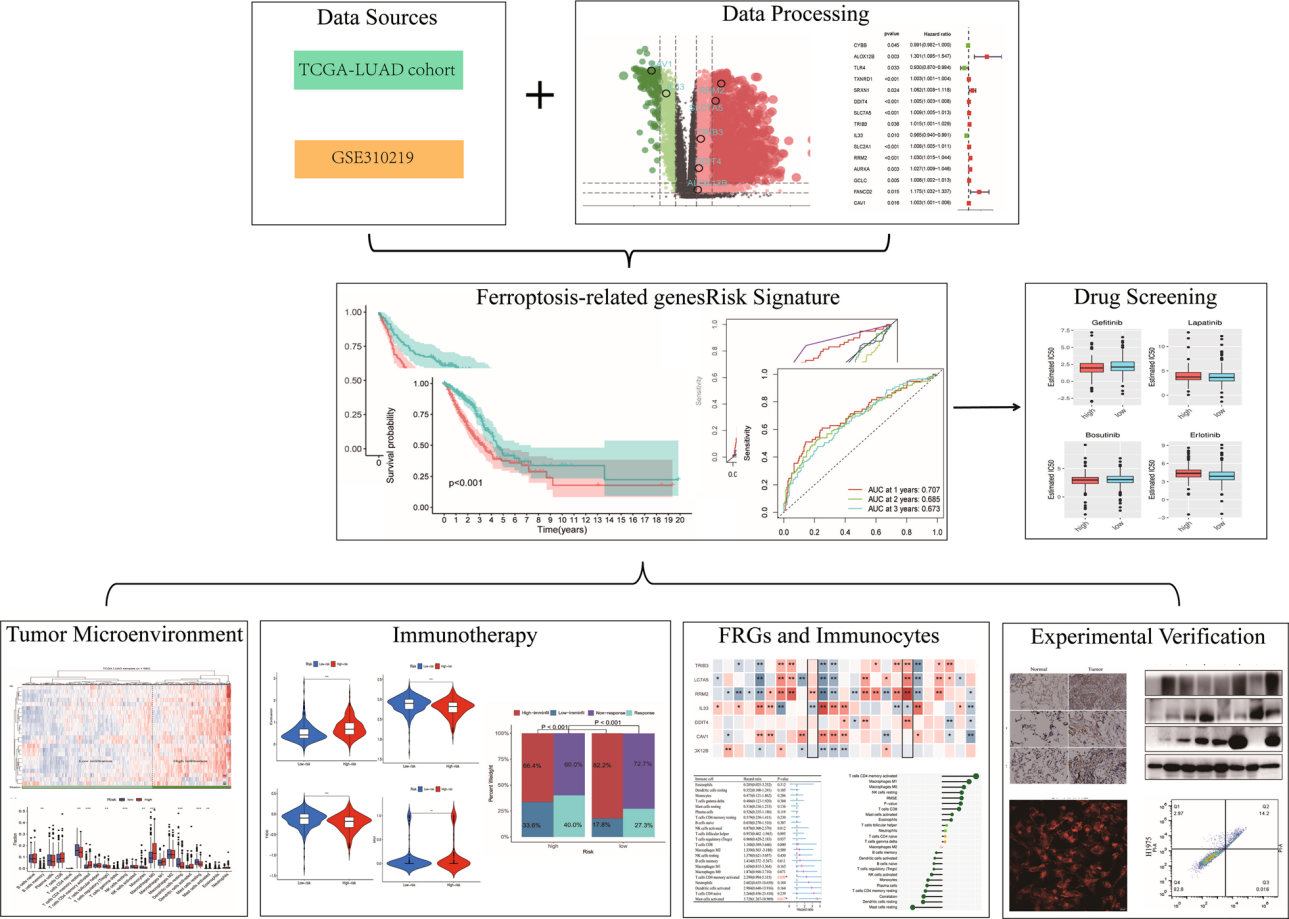
The flowchart of this study

Recent studies have shown that ferroptosis is closely related to many diseases, such as cancer, blood diseases, neurological diseases, kidney diseases, and local ischemia–reperfusion injury [15]. A growing number of studies investigating ferroptosis in cancer have revealed its potential as an immunotherapy strategy [16]. LUAD is the most common pathological type of non-small cell lung cancer, and most patients are diagnosed at advanced stages, losing their opportunities for surgical treatment. Chemotherapy, radiotherapy, and traditional Chinese medicine are common treatment choices for patients with advanced LUAD. However, the 5-year survival rate is only 15% [17]. The benefits of targeted therapy and immunotherapy in the treatment of advanced cancer patients have begun to change the treatment of cancer, providing new treatment methods for patients with advanced tumors. In this study, the seven-gene risk signature we established was closely related to the immune microenvironment of LUAD, and the immunocyte infiltration and immune function were significantly different in the high- and low-risk groups. Moreover, the two groups of patients with LUAD with high and low infiltration (revealed by immunocyte infiltration clustering) had different ferroptosis gene clusters and risk scores, which suggests that our seven-gene signature may provide guidance for LUAD immunotherapy. The immunotherapy analysis showed that patients with higher risk scores were more likely to benefit from immunotherapy. However, previous results showed that the overall infiltration level of immunocytes in high-risk patients was lower, perhaps because low immunocyte infiltration levels are related to a good prognosis, whereas increased infiltration levels of activated mast cells and activated CD4 memory T cells lead to a worse prognosis in patients with LUAD. Ultimately, patients with LUAD with lower infiltration levels were identified as high-risk by the seven-gene signature. Immunotherapy drugs can also activate the immune system to a greater extent; increase the infiltration of immunocytes related to a good prognosis and inhibit the activation of mast cells, CD4 memory T cells, and dendritic cells; and eventually improve the response rate to immunotherapy. These results show that FRGs regulate the tumor microenvironment of LUAD and influence the efficacy of immunotherapy. Gene mutation analysis in LUAD samples showed that seven FRGs had some degree of mutations; RRM2 and ALOX12B mutations were the most significant. Further analysis also found that the expression level of RRM2 was strongly correlated with the level of immunocytes in LUAD. In addition, we found that silencing RRM2 promoted elevated ferrous ion levels and lipid peroxidation accumulation and induced ferroptosis in LUAD. Therefore, we suggest that RRM2 may play a key role in LUAD (Fig. 10).

The inhibition of RRM2 reduces the level of dNTP, which is the basis for stable senescence-related cell growth arrest induced by oncogenes [18].Knocking down RRM2 significantly decreased proliferation during the S phase of the cell cycle [19, 20].Interestingly, researchers found that RRM2 regulated the expression of BCL-2 (a key determinant of cell apoptosis).Rahman et al.’s study found that RRM2 consumption significantly reduced the expression of Bcl-2 protein [21], and Jin et al. found that by overexpressing RRM2, the activity of the Bcl-2 signaling pathway was increased and the activity of the p53 signaling pathway was decreased [22]. These findings suggest that RRM2 plays an important role in the S phase during DNA replication, which may have potential therapeutic significance. Zhong et al. and Li et al. studied the potential of RRM2 as a therapeutic target for gastric cancer and glioblastoma [23, 24]. Another study found that human papillomavirus E7 oncoprotein increased the expression of RRM2 to promote angiogenesis in cervical cancer and that inhibiting RRM2 activity may be a new therapeutic strategy for human cervical cancer [25]. Most importantly, treatment methods related to RRM2 have greatly improved. Shao et al. found that RRM2 inhibitor could treat breast cancer [26, 27]. Another study showed that using gambogic acid to reduce the expression of RRM2 could improve the efficacy of gemcitabine in pancreatic cancer; gambogic acid caused pancreatic cancer cells to become sensitive to gemcitabine in vitro and in vivo by inhibiting the activation of the ERK/E2F1/RRM2 signaling pathway [28].

To date, few studies have explored the role of RRM2 in lung cancer. RRM2, which is highly expressed in lung cancer and related to a poor prognosis, regulates the immune microenvironment of LUAD, which is consistent with our results [10]. Cai et al. detected significant changes in RRM2 methylation in patients with non-small cell lung cancer using bioinformatics and found a significant correlation between RRM2 expression and multiple immunocyte infiltration [29]. Knockdown of RRM2 suppressed tumor growth in xenograft tumor models, and RRM2 deficiency increased CD8 + T cells in tumor tissues and the spleen [30].Bufu Tang et al. found that the expression level of RRM2 was positively correlated with neutrophil and macrophage infiltration in LUAD tissue, suggesting that RRM2 promoted lung cancer progression and affected macrophage infiltration, stimulated M1 phenotype polarization, and inhibited the M2 phenotype [10].

Our research has several limitations. First, our risk signature was built and validated using TCGA and the GEO database; no further external validation was performed with real-world prospective LUAD RNA-seq cohorts. Second, when analyzing the efficacy of immunotherapy, the LUAD sample size of the GSE126044 cohort was relatively small. Although positive results were obtained, their accuracy remains unclear. Therefore, we are attempting to find or establish a better and larger immunotherapy cohort of patients with LUAD. Third, although we identified a key role of RRM2 in LUAD using bioinformatics technology, we have not yet verified how RRM2 affects the development of LUAD by regulating mast cells and CD4 memory T cells or other mechanisms. Fourth, we screened drugs for high-risk patients with LUAD through the CMap and GDSC databases, but the specific efficacy and mechanism need to be further explored by cellular and animal experiments as well as clinical trials. Overall, more clinical samples and prospective experiments are necessary to study the mechanism by which RRM2 influences the LUAD immune microenvironment and immunotherapy.

## Conclusions

We established a risk prediction signature based on FRGs and found that it was closely associated with the immune microenvironment of LUAD. The key risk gene RRM2 influenced the prognosis of LUAD by regulating the infiltration of activated mast cells and activated CD4 memory T cells. Moreover, FRG-based LUAD clustering was associated with the risk signature and immune cell infiltration. In other words, the FRG clustering we provided may indicate potential LUAD immune subtypes. Furthermore, a correlation analysis supported the relationship between the signature and LUAD immunotherapy. In addition, our results provide a new perspective that FRGs, including RRM2, influence anti-PDL1 immunotherapeutic responses by regulating immune checkpoint gene expression and infiltration of immune cells in the tumor microenvironment. Finally, our results suggest that RRM2 is an important biomarker affecting the long-term survival of patients with LUAD, and silencing RRM2 could promote the occurrence of ferroptosis in lung cancer cells. Taken together, our results offer novel insights into the research and treatment of LUAD.

## Supplementary Information

**Additional file 1: Figure S1.** Ferroptosis-related clustering and seven FRGs affecting LUAD prognosis. (A, B, C, D) TCGA-LUAD cohort was divided into four clusters based on 70 differentially expressed FRGs. (E)Significant differences in OS between the 4 FRGs clusters. (F-L) Different expression levels of 7 key FRGs represent different OS in LUAD patients.

**Additional file 2: Figure S2.** Enrichment analysis. (A) KEGG enrichment, fluid shear stress and atherosclerosis, HIF-1 signaling pathways, lipid and atherosclerosis, and ferroptosis are enriched. **(**B) GSEA enrichment, small cell pulmonary carcinoma, melanoma, cancer pathway, P53 signaling pathway, pathogenic Escherichia coli infection were significantly enriched in high-risk patients. (C) GO enrichment.

**Additional file 3: Figure S3.** Clinical correlation heat map and the differential infiltration level of immunocytes in LUAD patients. (A)The proportion of four clinical stages in high- and low-risk groups. (B)Distribution of several clinical features between high- and low-risk groups. (C)The proportion of risk scores of four FRGs clusters. (D)The infiltration ratio of 22 immunocytes in each TCGA-LUAD sample. (E)Infiltration of 10 immunocytes in high- and low-risk groups. (F)The change trend of infiltration level of Mast cells resting with ALOX12B expression level.

**Additional file 4: Figure S4.** The infiltration level of immune cells reflects the OS of LUAD. (A-L) Kaplan–Meier curve. Different infiltration levels of 12 immunocytes correspond to different overall survival rates in LUAD patients.

**Additional file 5: Figure S5.** FRGs are involved in immune response and regulate the expression of immune checkpoints. (A)The change trend of TMB with RiskScore. (B)Different levels of TMB represent significantly different OS in LUAD patients. (C, D) Different ImmunScore and StromalScore represent significantly different OS in LUAD patients. (E) Mutation analysis of 7FRGs in TCGA-LUAD cohort. (F) The difference of RRM2 expression between LUAD patients with two different immunotherapy responses. (G)The difference of immunocytes infiltration and response to anti-PDL1 immunotherapy between high- and low-risk patients. (H)Relationship between PD1 expression and 15 prognostic FRGs. (I) Relationship between PDL1 expression and 15 prognostic FRGs.

**Additional file 6: Figure S6.** Six antitumor drugs with significantly lower IC50 and six chemical structures of the top molecules for high-risk LUAD patients selected from CMAP database. (A)Bosutinib.(B)JNK.Inhibitor.VIII.(C)Docetaxel.(D)Tipifarnib.(E)Dasatinib.(F)Gefitinib.(G)Medrysone. (H)phenoxybenzamine. (I)Vorinostat. (J)Thioguanosine. (K)apigenin. (L)chrysin.

## Data Availability

The data used to support the findings of this study are included within the article.
