# Supplementary figures and images for: Identification and validation of a ferroptosis-related gene to predict survival outcomes and the immune microenvironment in lung adenocarcinoma
Source: Cancer Cell Int. 2022 Sep 24;22:292. doi: 10.1186/s12935-022-02699-4 (PMC9508770; doi:10.1186/s12935-022-02699-4)

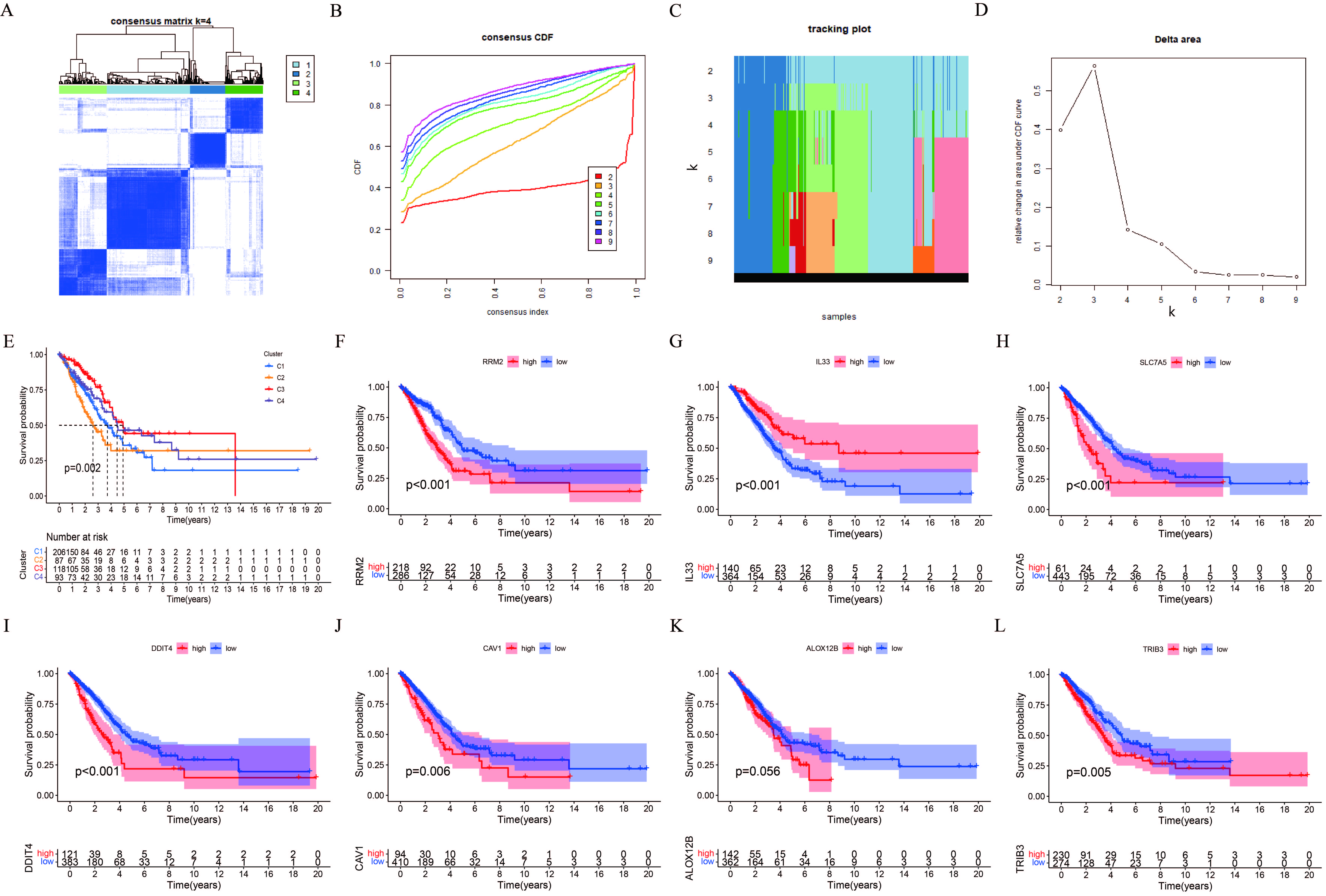

Supplement: Supplementary file 1 — Additional file 1: Figure S1. Ferroptosis-related clustering and seven FRGs affecting LUAD prognosis. (A, B, C, D) TCGA-LUAD cohort was divided into four clusters based on 70 differentially expressed FRGs. (E)Significant differences in OS between the 4 FRGs clusters. (F-L) Different expression levels of 7 key FRGs represent different OS in LUAD patients. [file 12935_2022_2699_MOESM1_ESM.jpg]

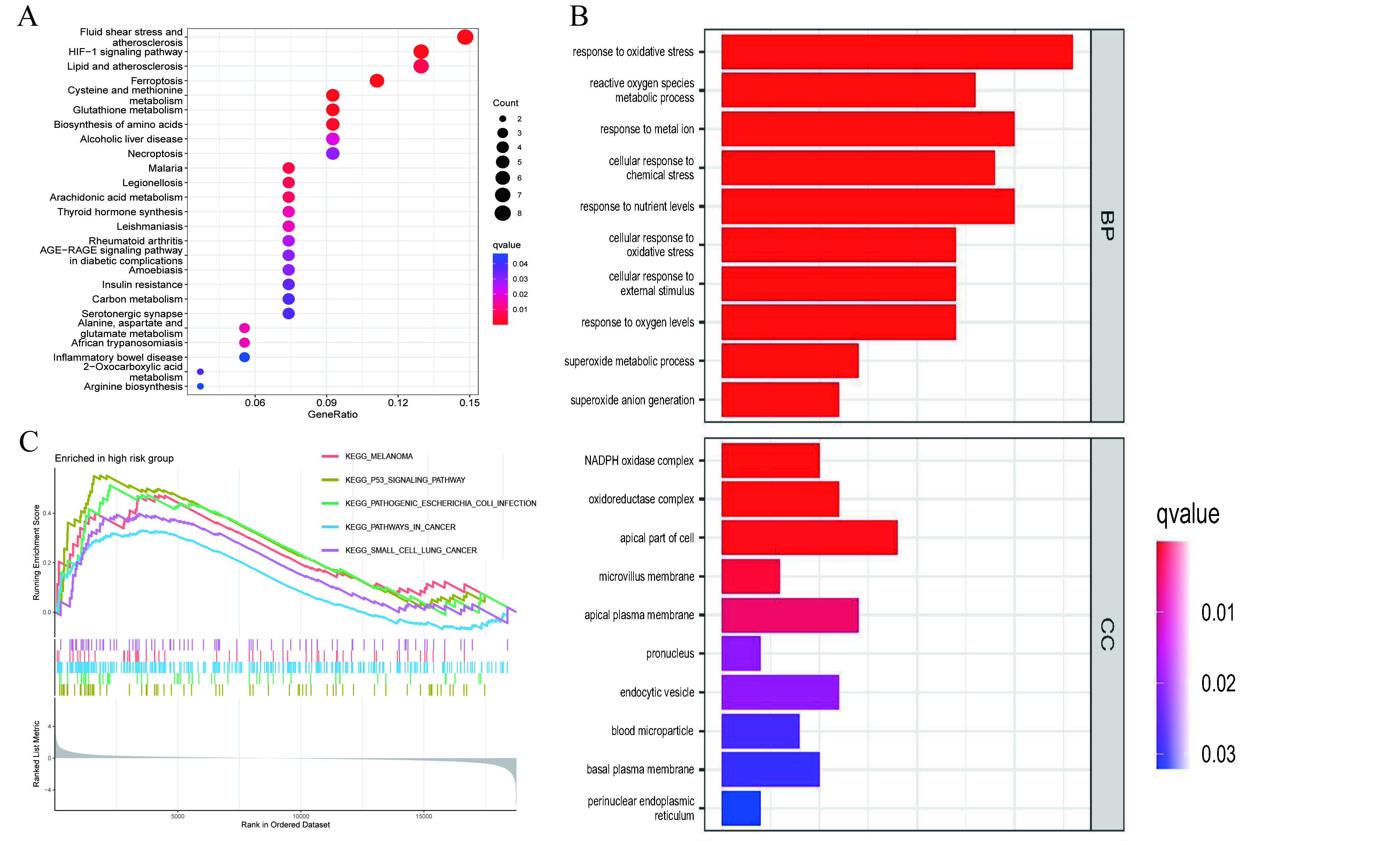

Supplement: Supplementary file 2 — Additional file 2: Figure S2. Enrichment analysis. (A) KEGG enrichment, fluid shear stress and atherosclerosis, HIF-1 signaling pathways, lipid and atherosclerosis, and ferroptosis are enriched. (B) GSEA enrichment, small cell pulmonary carcinoma, melanoma, cancer pathway, P53 signaling pathway, pathogenic Escherichia coli infection were significantly enriched in high-risk patients. (C) GO enrichment. [file 12935_2022_2699_MOESM2_ESM.jpg]

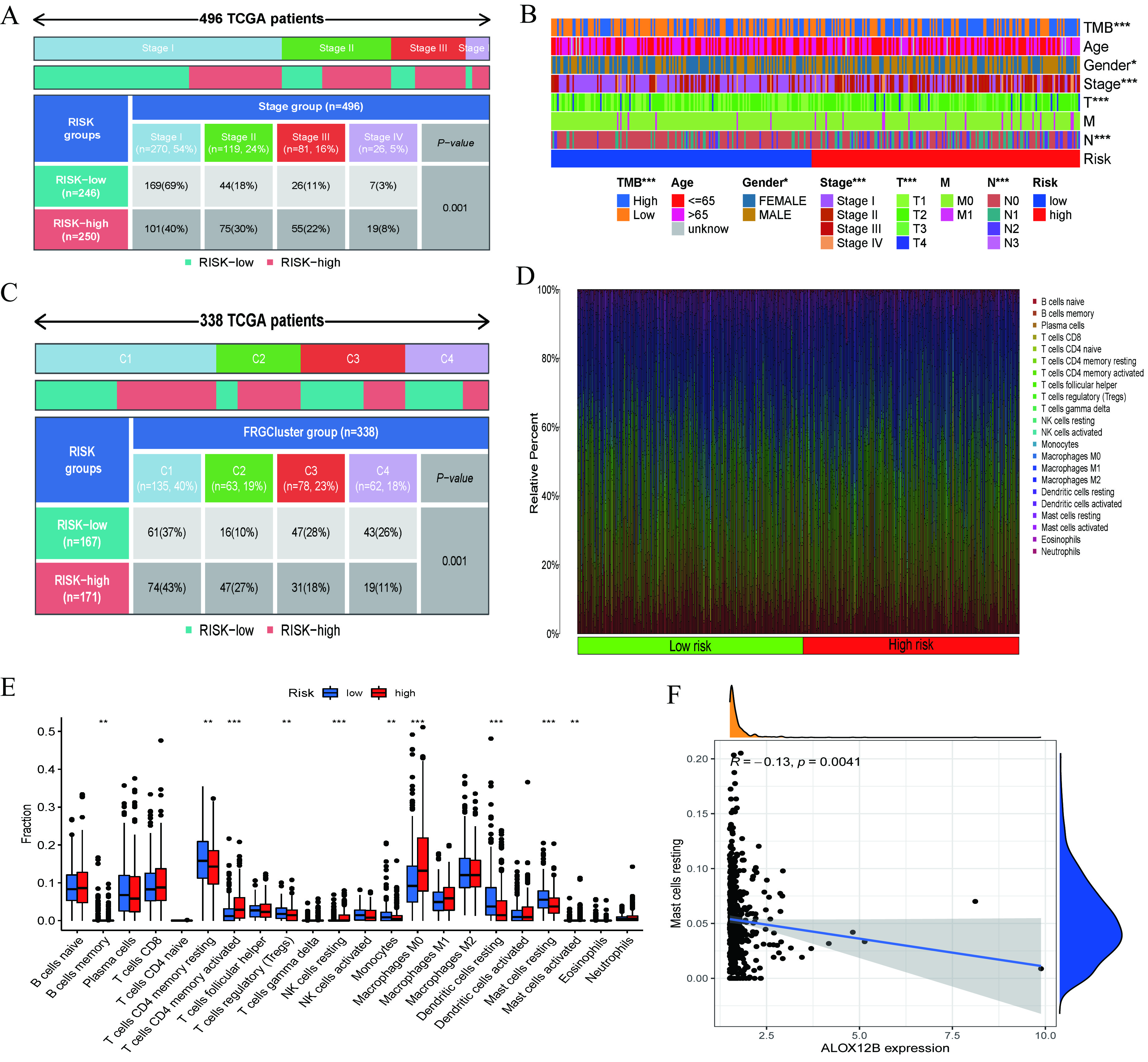

Supplement: Supplementary file 3 — Additional file 3: Figure S3. Clinical correlation heat map and the differential infiltration level of immunocytes in LUAD patients. (A)The proportion of four clinical stages in high- and low-risk groups. (B)Distribution of several clinical features between high- and low-risk groups. (C)The proportion of risk scores of four FRGs clusters. (D)The infiltration ratio of 22 immunocytes in each TCGA-LUAD sample. (E)Infiltration of 10 immunocytes in high- and low-risk groups. (F)The change trend of infiltration level of Mast cells resting with ALOX12B expression level. [file 12935_2022_2699_MOESM3_ESM.jpg]

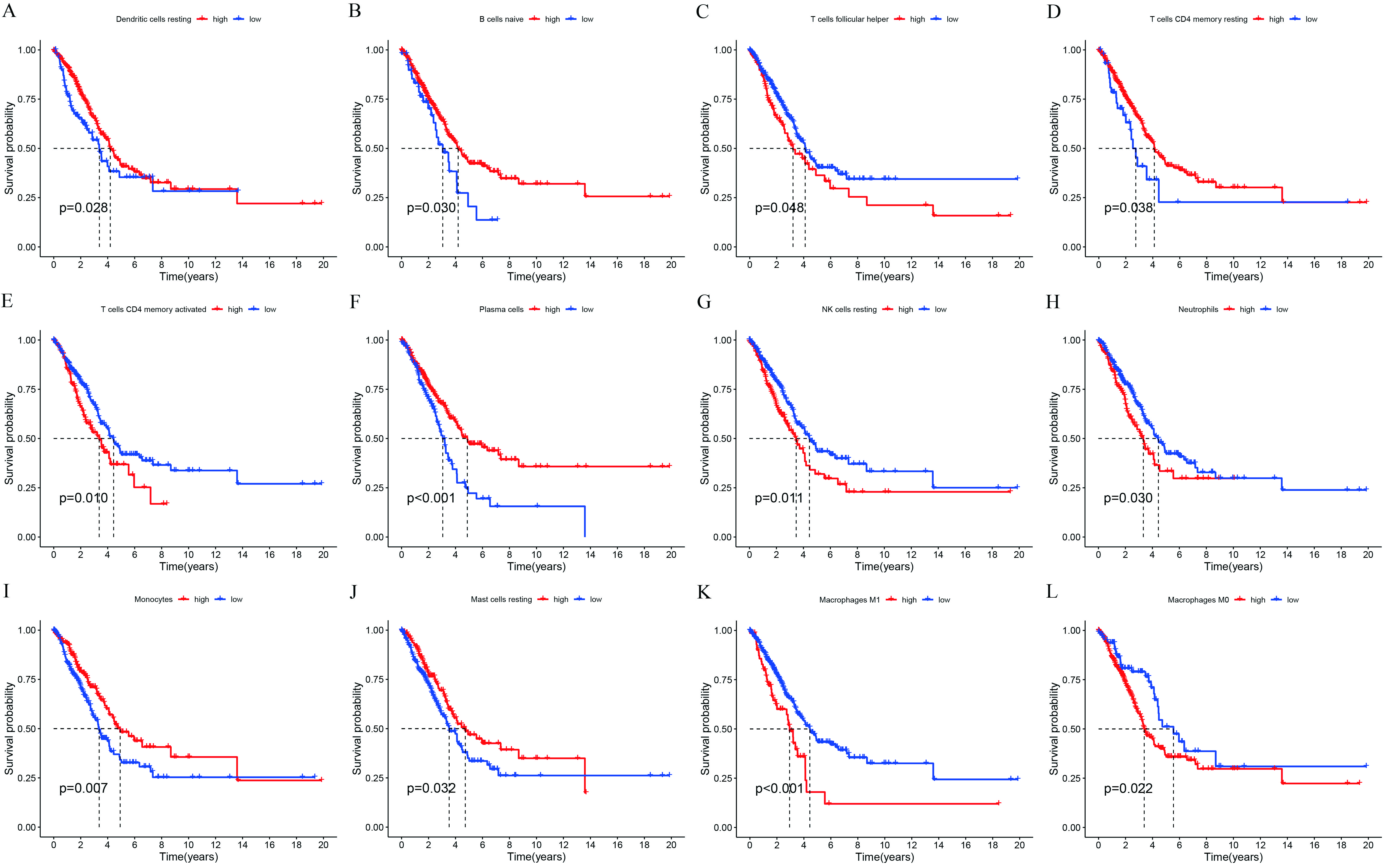

Supplement: Supplementary file 4 — Additional file 4: Figure S4. The infiltration level of immune cells reflects the OS of LUAD. (A-L) Kaplan–Meier curve. Different infiltration levels of 12 immunocytes correspond to different overall survival rates in LUAD patients. [file 12935_2022_2699_MOESM4_ESM.jpg]

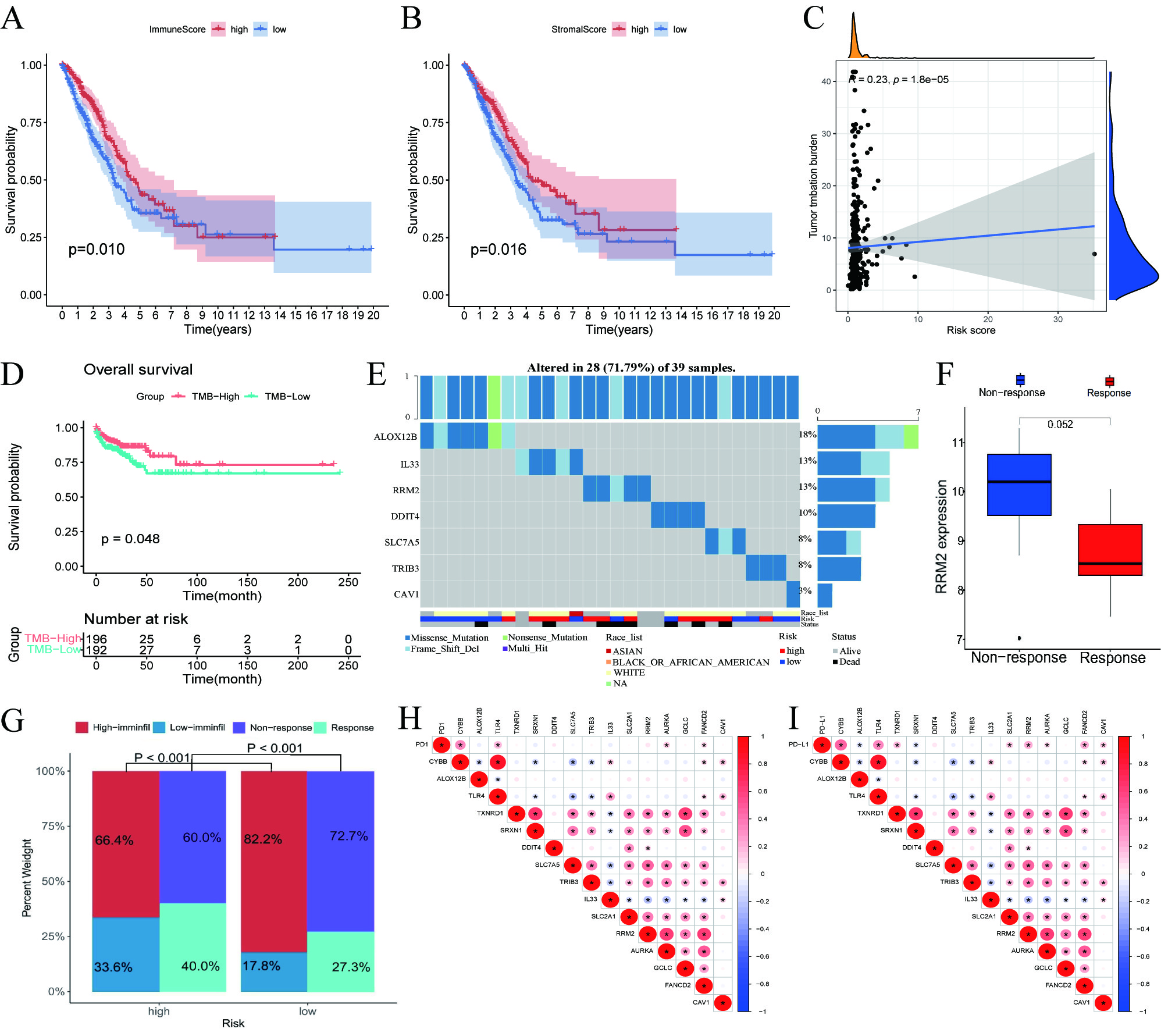

Supplement: Supplementary file 5 — Additional file 5: Figure S5. FRGs are involved in immune response and regulate the expression of immune checkpoints. (A)The change trend of TMB with RiskScore. (B)Different levels of TMB represent significantly different OS in LUAD patients. (C, D) Different ImmunScore and StromalScore represent significantly different OS in LUAD patients. (E) Mutation analysis of 7FRGs in TCGA-LUAD cohort. (F) The difference of RRM2 expression between LUAD patients with two different immunotherapy responses. (G)The difference of immunocytes infiltration and response to anti-PDL1 immunotherapy between high- and low-risk patients. (H)Relationship between PD1 expression and 15 prognostic FRGs. (I) Relationship between PDL1 expression and 15 prognostic FRGs. [file 12935_2022_2699_MOESM5_ESM.jpg]

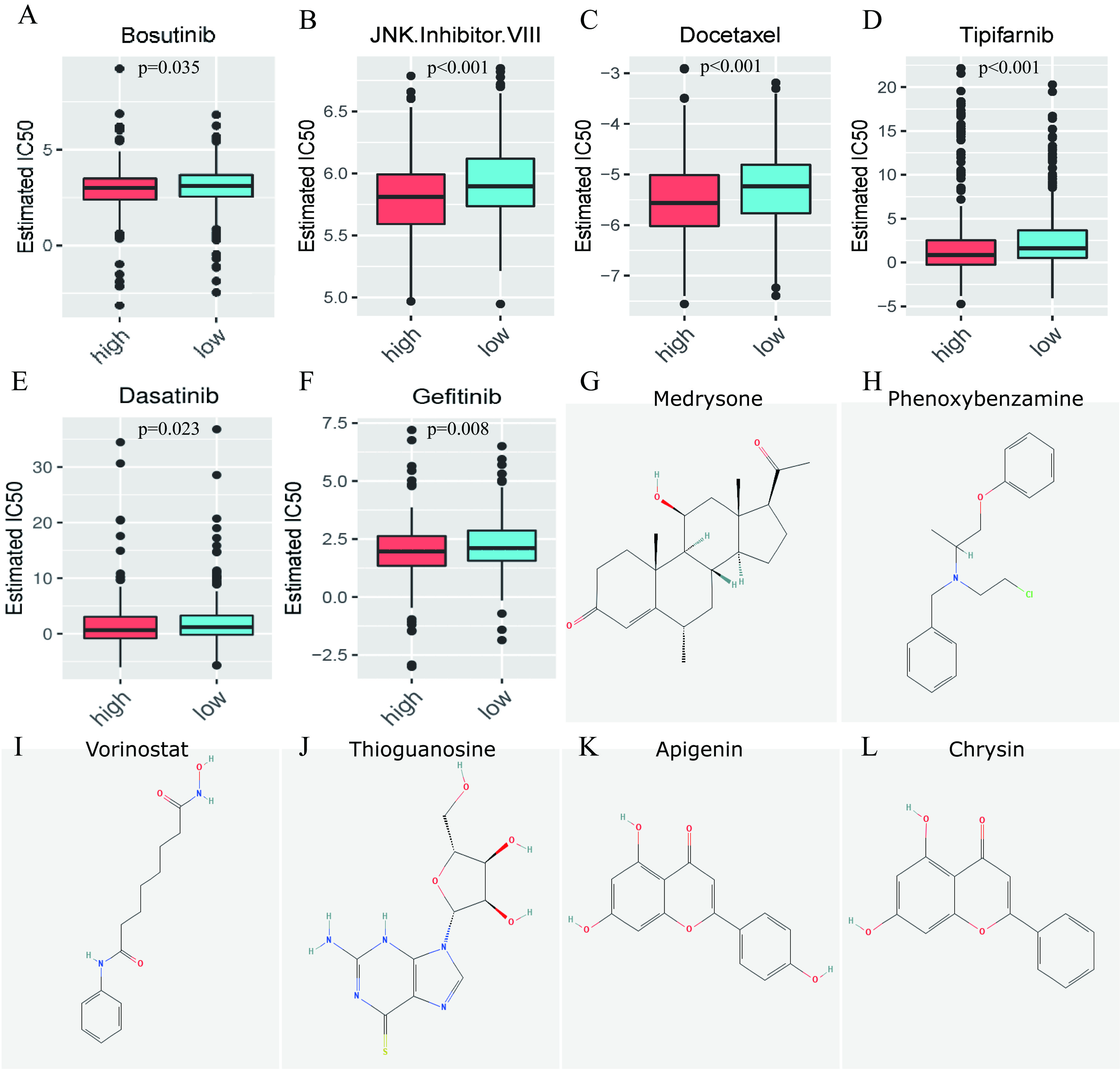

Supplement: Supplementary file 6 — Additional file 6: Figure S6. Six antitumor drugs with significantly lower IC50 and six chemical structures of the top molecules for high-risk LUAD patients selected from CMAP database. (A)Bosutinib.(B)JNK.Inhibitor.VIII.(C)Docetaxel.(D)Tipifarnib.(E)Dasatinib.(F)Gefitinib.(G)Medrysone. (H)phenoxybenzamine. (I)Vorinostat. (J)Thioguanosine. (K)apigenin. (L)chrysin. [file 12935_2022_2699_MOESM6_ESM.jpg]
